# Network Properties of the Ensemble of RNA Structures

**DOI:** 10.1371/journal.pone.0139476

**Published:** 2015-10-21

**Authors:** Peter Clote, Amir Bayegan

**Affiliations:** Department of Biology, Boston College, Chestnut Hill, MA 02467 United States of America; Ben-Gurion University, ISRAEL

## Abstract

We describe the first dynamic programming algorithm that computes the expected degree for the network, or graph *G* = (*V, E*) of all secondary structures of a given RNA sequence **a** = *a*_1_, …, *a*_*n*_. Here, the nodes *V* correspond to all secondary structures of **a**, while an edge exists between nodes *s, t* if the secondary structure *t* can be obtained from *s* by adding, removing or shifting a base pair. Since secondary structure kinetics programs implement the Gillespie algorithm, which simulates a random walk on the network of secondary structures, the expected network degree may provide a better understanding of kinetics of RNA folding when allowing defect diffusion, helix zippering, and related conformation transformations. We determine the correlation between expected network degree, contact order, conformational entropy, and expected number of native contacts for a benchmarking dataset of RNAs. Source code is available at http://bioinformatics.bc.edu/clotelab/RNAexpNumNbors.

## Introduction

RNA folding kinetics plays an important role in various biological processes, including *(i)* trans splicing of RNA, which is controlled by trypanosomal spliced leader (SL) RNA kinetics [1], and *(ii)* the *hok/sok* host-killing/suppression of killing (*hok/sok*) system that kills *E. coli* replicates if insufficient plasmids are transfered to the new daughter cell [2]. To better understand how macromolecules fold into their native state, energy landscapes for protein and RNA folding have been intensively studied [3–8]. In the case of RNA secondary structure formation, numerous algorithms have been developed beyond thermodynamic equilibrium structure prediction [9, 10], including algorithms (1) to determine optimal or near-optimal folding pathways, [6, 7, 11–13], (2) to compute explicit solutions of the master equation for possibly coarse-grained models [14–18], and (3) to simulate stepwise folding from an initial secondary structure to the target minimum free energy (MFE) structure [5, 19–24]. Nevertheless, RNA secondary structure folding kinetics remains a computationally difficult problem, since it is known that the problem of determining optimal folding pathways is NP-complete [25]. Despite increasing awareness of the importance of regulatory and catalytic RNA, no database currently exists of experimentally determined RNA folding rates, in contrast to the situation for proteins. Indeed, KineticDB is a database that provides users with a diverse set of experimentally determined folding rates for 87 unique proteins and approximately one hundred mutants [26].

It is currently an open problem to predict the folding rate of proteins and RNA molecules from the sequence alone. The goal of this paper is to raise awareness of this problem—in particular, the problem of predicting RNA secondary structure folding rate from the nucleotide sequence. For proteins, it has been shown that *absolute contact order*, which scales as ≈ *n* ^0.7^ for sequence length *n*, correlates rather well with protein folding rates for two- and multi-state folding proteins, reaching a correlation of 77% [27]—see as well Table 1 of [28]. Here, protein contact order is defined as the average chain separation of residues in contact (e.g. within 6 Å) in the native structure. It has also been shown that the number of native contacts correlates with folding rates of small single-domain proteins with two-state kinetics. In this case, Makarov et al. showed that ln(*k*) ≈ ln(*N*) + *a* + *bN*, where *k* denotes the folding rate, *N* is the number of contacts in the folded state, and *a, b* are constants whose physical meaning is understood [29].

**Table 1:** This table compares expected network degree and the length-normalized expected network degree for three RNA sequences of moderate size: 32 nt *fruA*, encoding the A subunit of coenzyme F420-reducing hydrogenase; tRNA RA1180, 56 nt spliced leader RNA from *L. collosoma*; 76 nt transfer RNA with accession code RA1180 from the database tRNAdb 2009 [41]. *Unif-MS1* [resp. *Unif-MS2*] denote the expected network degree for model B (uniform probability) for MS1 [resp. MS2] move set. *Turner99-MS1* [resp. *Turner99-MS2*] and *Turner04-MS1* [resp. *Turner04-MS2*] and denote the expected network degree for model C (Boltzmann probability for Turner 1999 and Turner 2004 energy parameters [36]) for MS1 [resp. MS2] move set. *Sample-MS1* [resp. *Sample-MS2*] denotes the *approximation* of the expected network degree for model C (Turner 1999 and Turner 2004 parameters) obtained by generating low energy structures by RNAsubopt -d0 -e 12, as explained in the text. In the case of *fruA*, all 971,399 possible structures were generated by RNAsubopt -d0 -e 100, so that *Sample-MS1* and *Sample-MS2* values are correct—for this reason, the standard deviation values are not included. Note that for *L. collosoma*, the expected degree values for the Turner 2004 energy parameters are *much* larger than those obtained for Turner 1999 energy parameters.

Unnormalized									
	len	Unif-MS1	Unif-MS2	Turner99-MS1	Turner04-MS1	Turner99-MS2	Turner04-MS2	Sample-MS1	Sample-MS2
fruA	32	10.66	27.60	10.00	9.98	13.03	13.07	10.08	13.13
L. collosoma	56	20.47	52.64	48.37	70.03	69.26	93.58	69.87 ± 34.04	90.46 ± 37.71
tRNA	76	28.22	71.59	26.27	26.10	35.43	37.59	29.11 ± 4.63	46.51 ± 8.74
Normalized									
	len	Unif-MS1	Unif-MS2	Turner99-MS1	Turner04-MS1	Turner99-MS2	Turner04-MS2	Sample-MS1	Sample-MS2
fruA	32	0.3330	0.8624	0.3125	0.3120	0.4072	0.4084	0.3150	0.4103
L. collosoma	56	0.3655	52.6355	0.8637	1.2505	1.2368	1.6710	1.2477 ± 0.6079	1.6153 ± 0.6734
tRNA	76	0.3713	71.5946	0.3457	0.3434	0.4662	0.4946	0.3830 ± 0.0610	0.6120 ± 0.1150

To our knowledge, no relation has been established between RNA folding rate and either contact order or the number of native contacts, due in part to the above-mentioned absence of a database of RNA folding rates, and due in part to the notorious difficulty of estimating RNA secondary structure folding rates when using secondary structure kinetics software such as Kinfold [5], Kinefold [20], RNAKinetics [21], KFold [30], or other software [22, 23]. Such programs implement an event-driven Monte Carlo algorithm known as Gillespie’s algorithm [31]; it follows that repeated (time-consuming) simulations will generate a collection of mean first passage times which are approximately exponentially distributed. Since an exponential distribution has the property that the mean is equal to the standard deviation, it follows that precise kinetics obtained by such methods necessarily requires inordinate computation time (e.g. the population occupancy curve for yeast phe-tRNA required 3 months of CPU time on a 2.4 GHz Intel Pentium 4 running linux [14]). Until the availability of a database of experimentally determined RNA folding rates, it is likely that the best approximation of folding rates can be made using exact, coarse-grained approaches using spectral methods, as Treekin [14], basin hopping with RNAlocmin [17], and Hermes [18].

**Fig 1:**
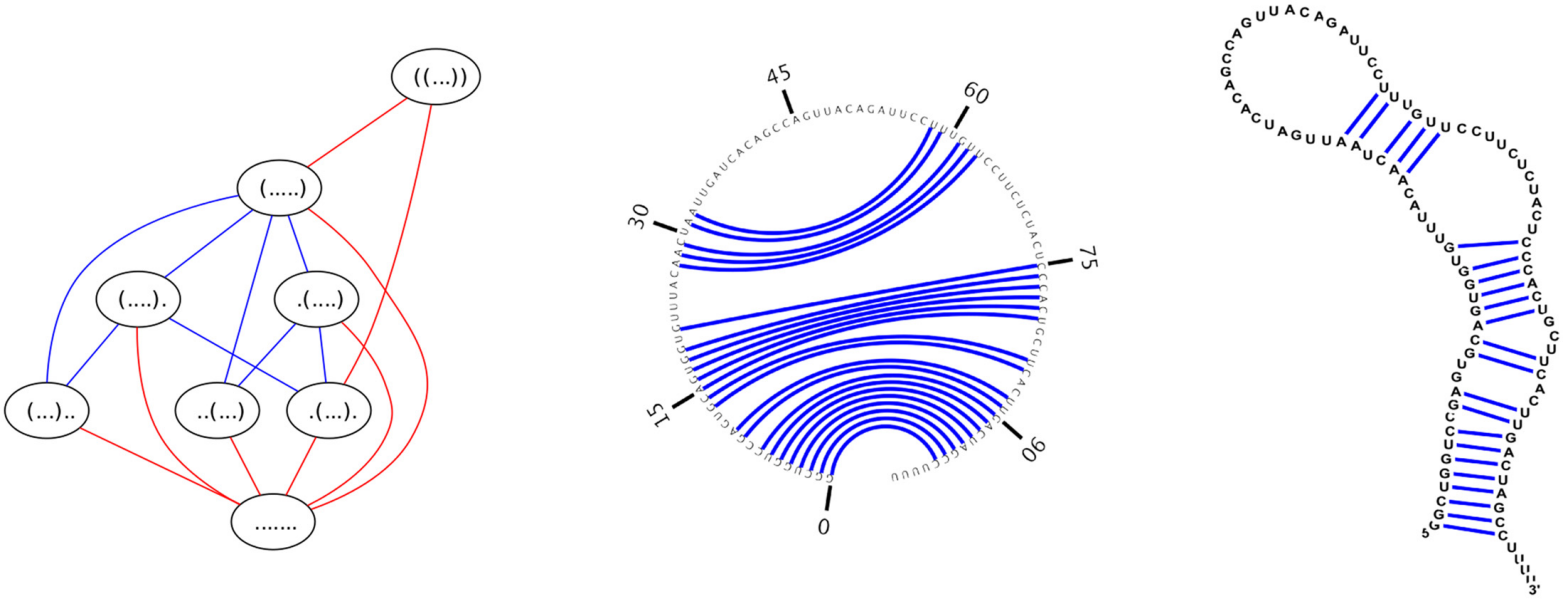
*(Left)* Network for the toy 7-mer GGGGCCC which has 8 nodes and 16 edges (hence 32 directed edges). The expected network degree is $\frac{32}{8}=4$. Red edges indicate base pair addition or removal, while blue edges indicate shift moves. *(Center)* Feynman circular representation of secondary structure of Y RNA. *(Right)* Conventional representation of secondary structure of Y RNA. According to [55], one function of Y RNA is to bind to certain misfolded RNAs, including 5S rRNA, as part of a quality control mechanism. The secondary structure depicted is the consensus secondary structure of Y RNA with EMBL access number AAPY01489510:220–119 from Rfam family RF00195 in the Rfam database [56]. Images produced with sofware jViz [57].

Apart from contact order and the number of native contacts, the *expected degree* of the network of RNA secondary structures of an RNA sequence is another order parameter that could play a role in RNA folding kinetics—see the left panel of Fig 1 for an example of expected network degree for the toy sequence GGGGCCC. Here, the degree of a node (secondary structure) *s* is the number of secondary structures *t* that can be obtained from *s* by the addition, removal or *shift* of a base pair. These moves constitute the default move set employed by the program Kinfold [5], often used to estimate RNA folding kinetics. Moreover, by analyzing the network *G* = (*V, E*), whose node set *V* consists of low energy secondary structures of *E. coli* phe-tRNA (RF6280 [32]) and whose edge set *E* consists of directed edges *s* → *t*, where *t* is obtained from *s* by a base pair addition, removal or shift, the network for phe-tRNA was shown to be *small-world* in [33].

In this paper, we provide the first algorithm to efficiently compute the expected degree of an RNA network of secondary structures. Our work generalizes a recent paper [34], which describes a vastly simpler algorithm to compute the expected degree without consideration of shift moves. Since our current algorithm is surprisingly complex, for clarity of exposition, we consider three successive models. Model A is the RNA *homopolymer* model [35], in which any two positions *i, j* can constitute a base pair, provided only that *i* + 1 < *j*. Model B is the usual RNA secondary structure model, where positions *i, j* can constitute a base pair if the corresponding nucleotides form a Watson-Crick or wobble pair and *i* +3 < *j*; however, in Model B, the energy of a structure is taken to be zero, so the probability of a structure is simply one over the number of structures. Model C extends Model B by using the Turner 2004 energy parameters [36] without dangles. Our algorithms have been extensively tested against brute-force exhaustive methods to be sure of algorithm and implementation. Finally, we begin a preliminary investigation into the relation between network degree, contact order, conformational entropy, and number of native contacts using two benchmarking sets of RNA structures. Since we show later that expected network degree is linear in sequence length for the (theoretical) homopolymer case, we additionally compute the length-normalized network degree.

### Preliminaries

**Definition 1**. *A secondary structure for a given RNA nucleotide sequence a_1_*, …, *a*_*n*_ *is a set s of base pairs (i, j), where* 1 ≤ *i* < *j* ≤ *n*, *such that*:

1. *if (i, j)* ∈ *s then a_i_, a_j_ form either a Watson-Crick (AU, UA, CG, GC) or wobble (GU, UG) base pair*,
2. *if (i, j)* ∈ *s then j* − *i* > *θ* = 3 *(a steric constraint requiring that there be at least θ* = 3 *unpaired bases between any two positions that are paired)*,
3. *if (i, j*) ∈ *s then for all i*′ ≠ *i and j*′ ≠ *j, (i*′, *j*) ∉ *s and (i, j*′) ∉ *s (nonexistence of base triples)*,
4. *if (i, j)* ∈ *s and (k, ℓ) ∈ s, then it is not the case that i < k < j < ℓ (nonexistence of pseudoknots)*.

Secondary structures can be depicted in several equivalent manners. For instance, the sequence and dot bracket representation for the secondary structure of Y RNA with EMBL access number AAPY01489510:220–119 is given by

GGCUGGUCCGAGUGCAGUGGUGUUUACAACUAAUUGAUCACAGCCAGUUACAGAUUCCUUUGUUCCUUCUCUACUCCCACUGCUUCACUUGACUAGCCUUUU ((((((((.((..(((((((.(.....(((.((.........................)).)))...........))))))...))..))))))))))....

Y RNA is a noncoding RNA, known to be required for the initiation of chromosomal DNA replication in mammalian cells [37]; a distinct function of Y RNA is mentioned in the caption to Fig 1, where two other formats for this secondary structure are depicted. A base pair (*i, j*) of structure *s* is an *external* base pair, if there is no base pair (*x, y*) ∈ *s* with the property that *x* < *i* < *j* < *y*. A position 1 ≤ *k* ≤ *n* is said to be *visible* in *s* if there is no base pair (*i, j*) ∈ *s* with the property that *i* ≤ *k* ≤ *j*. The secondary structure of Y RNA in Fig 1 has only one external base pair, i.e. (1, 98), and only four visible positions, i.e. positions 99, 100, 101, 102. Throughout the remainder of this paper, *structure* will mean secondary structure.

The base pair distance *d*_BP_(*s, t*) between secondary structures *s, t* is the number of base pairs ∣*s* − *t*∣ + ∣*t* − *s*∣ belonging to *s* but not *t*, or vice versa. A shift move from base pair (*i, j*) in the structure *s* is of the form (*i, k*) [resp. (*k, j*)], where (*s* \ {(*i, j*)}) ∪ {(*i, k*)} [resp. (*s* \ {(*i, j*)}) ∪ {(*k, j*)}] is a valid secondary structure. Throughout, let *bp*(*i, j*) be a boolean valued function, where *bp*(*i, j*) = 1 if positions *i, j* can form a base pair; i.e. if *a*_*i*_, *a*_*j*_ constitute a Watson-Crick or wobble pair. Reference [5] describes the Kinfold program, which implements the Gillespie algorithm [31] for RNA secondary structure folding kinetics. Kinfold produces secondary structure folding trajectories, or sequences *s* = *s*_0_, *s*_1_, …, *s*_*m*_ = *t*, where for 0 ≤ *i* < *m, s*_*i*+1_ is obtained from *s*_*i*_ by the addition or deletion of a base pair, and (optionally) by a shift move. These are defined as follows.

**Fig 2:**
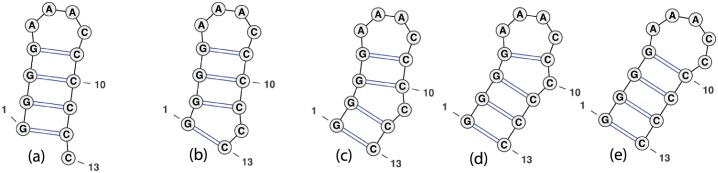
Defect diffusion [38], where a bulge migrates stepwise to become absorbed in an hairpin loop. The move from structure (a) to structure (b) is possible by the shift (1, 12) → (1, 13), the move from (b) to (c) by shift (2, 11) → (2, 12), etc. Our algorithm properly accounts for such moves with respect to energy models A, B, C. Image adapted from figure on page 26 [19] and produced by VARNA [58].

**Fig 3:**
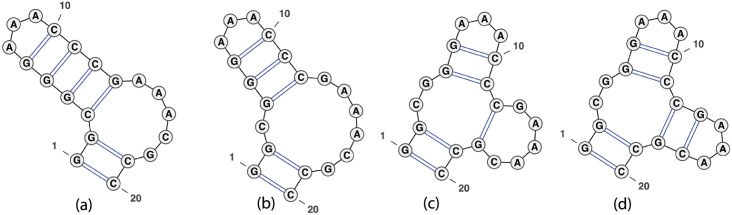
Example of multiloop creation which is handled by our algorithm for all energy models, including the Turner energy model. To move from (a) to (b), remove the base pair (3, 13); to move from (b) to (c), shift (4, 12) → (12, 18); to move from (c) to (d), add base pair (13, 17). Image produced by VARNA [58].

**Fig 4:**
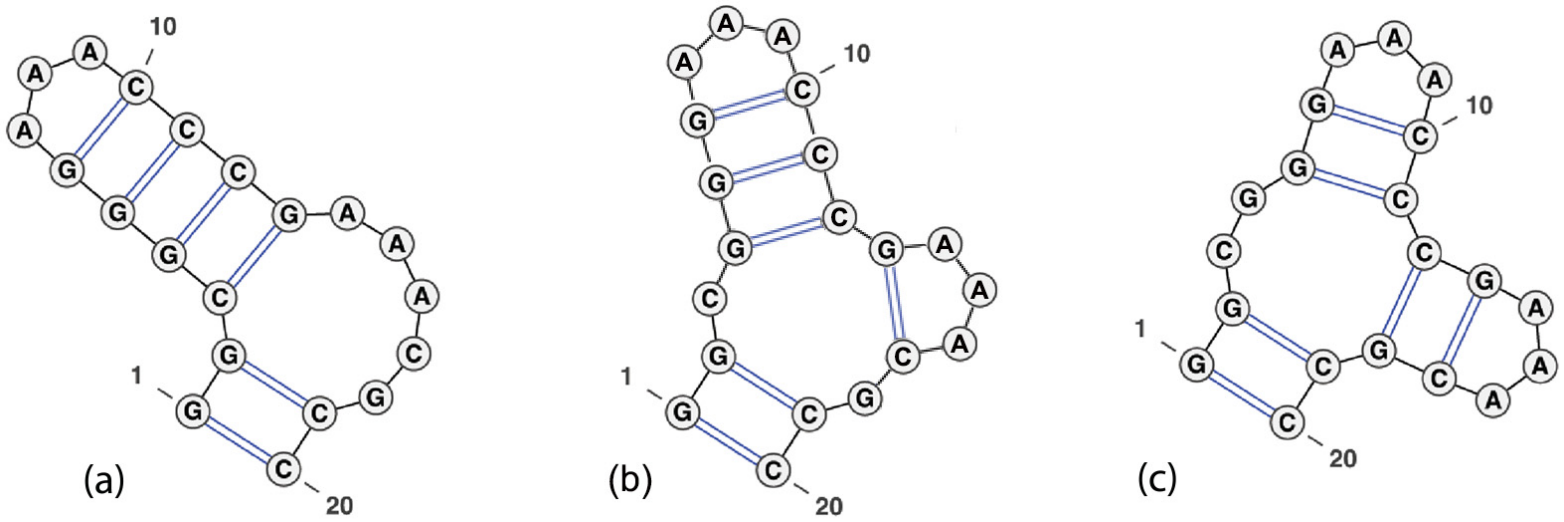
Example of multiloop creation which is handled by our algorithm for energy models A, B but not for Turner energy model C. To move from (a) to (b), apply the shift (3, 13) → (13, 17); to move from (b) to (c), apply the shift (4, 12) → (12, 18). Our algorithm for the Turner energy model properly treats the move from (a) to (b), but not from (b) to (c), as explained in the Remark at the end of Section “Remaining recursions for *Q*_*i*,*j*_ and *Z*_*i*,*j*_”. Image adapted from figure on page 27 [19] and produced by VARNA [58].

The move set MS1 allows a move from structure *s* to structure *t*, if *t* can be obtained from *s* by the removal of addition of a base pair; i.e. if *t* = *s* \ {(*i, j*)} or *t* = *s* ∪ {(*i, j*)}. The move set MS2 allows moves from MS1 as well as four shift moves, described by the following. Structure *t* is obtained from *s* by the replacement of base pair (*i, j*) ∈ *s* by the distinct base pair (*i, j*′), or (*j*′, *i*), or (*i*′, *j*), or (*j, i*′), provided that *t* is a valid secondary structure. Figs 2, 3 and 4 depict some typical shift moves, including *defect diffusion* [38].

### Expected network degree

Throughout this paper, let **a** = *a*_1_, …, *a*_*n*_ be a fixed, but arbitrary RNA sequence. Consider the set of all secondary structures of **a** as a network, or graph, where two structures *s, t*, are connected by an edge if *t* can be obtained from *s* by a base pair addition, removal or shift.

**Fig 5:**
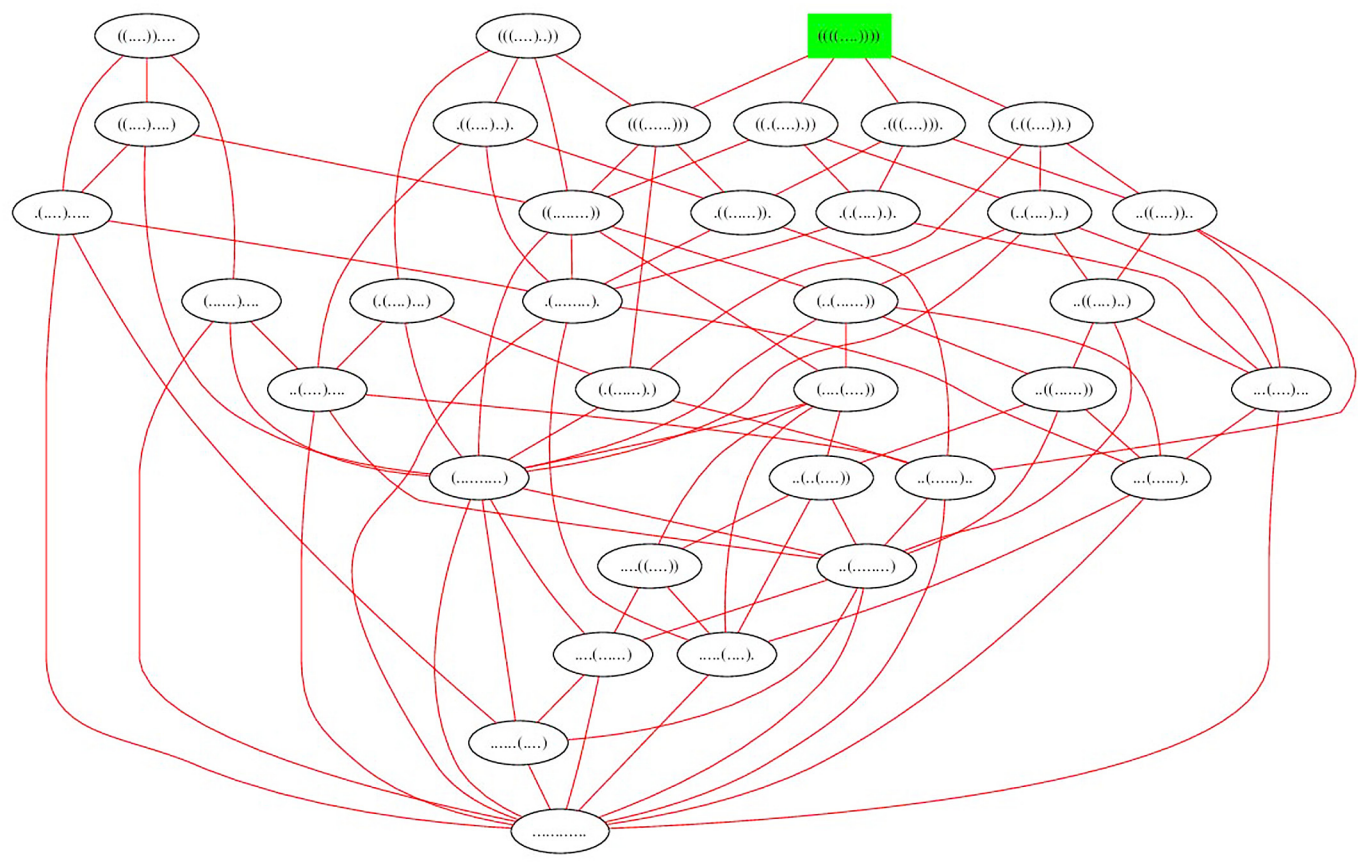
The network of all secondary structures of the 12 nt (toy) sequence ACGUACGUACGU. The minimum free energy structure is shown in green. Edges connect structures *s, t*, such that *t* is obtained by a move in MS2 from *s*, or vice versa; i.e. structures are connected by an edge if they differ by a base pair addition, removal or shift. There are 35 structures, 126 edges between structures that differ by a base pair removal or addition, and 68 edges between structures that differ by a base pair shift. Altogether, there are 194 edges. It follows that the average network degree is $\frac{194}{35}=5.54$.

**Fig 6:**
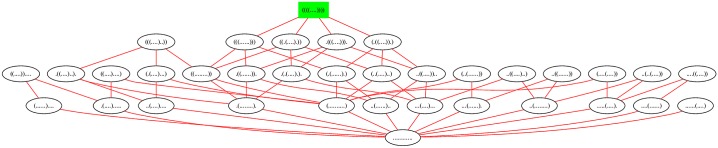
The network of all secondary structures of the 12 nt sequence ACGUACGUACGU, where edges connect structures *s, t*, such that *t* is obtained by a move in MS1 from *s*, or vice versa; i.e. structures are connected by an edge if they differ by a base pair addition or removal. There are 35 structures, 126 edges between structures that differ by a base pair removal or addition, hence the average network degree is $\frac{126}{35}=3.6$.

**Fig 7:**
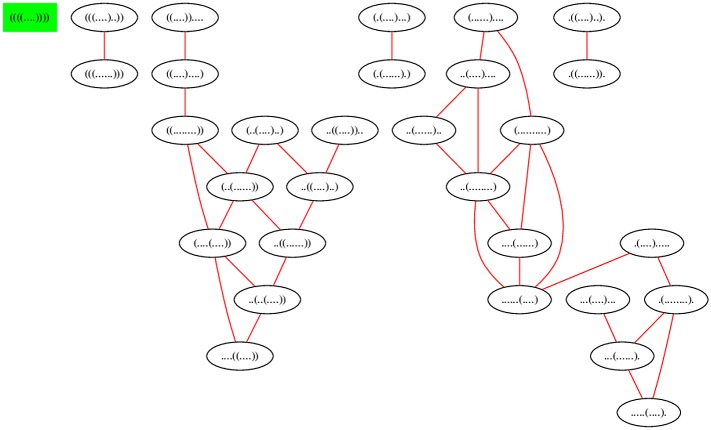
The network of all secondary structures of the 12 nt sequence ACGUACGUACGU, where edges appear between structures that differ by a shift move. There are 35 structures, 68 edges between structures that differ by a base pair shift, hence the average network degree is $\frac{68}{35}=1.94$. Note that the network is not connected, unlike the previous two networks.

Fig 1 displays the network for a toy 7 nt sequence GGGGCCC, where moves come from move set MS2 (base pair additions and removals indicated by red edge; shift moves indicated by blue edge). Fig 5 displays the network for the slightly larger sequence ACGUACGUACGU, where moves come from move set MS2. In contrast, Fig 6 displays the network where moves are restricted to the move set MS1, and Fig 7 displays the network where shifts are the only allowable move—i.e. moves are restricted to the move set MS2\MS1. When moves are allowed to range over either MS1, or over MS2, the resulting network is connected; this is not the case for moves in MS2\MS1. Since the network represents intermediate moves in RNA folding trajectories, it is of interest to know the average network degree. This was done for move set MS1 in [34]. The goal of this paper is to describe the first algorithm, which computes the expected network degree, or equivalently, the expected number of neighbors, for the RNA network defined with move set MS2. Computing the expected number of neighbors when including shift moves turns out to be remarkably difficult, so for clarity of exposition, we present three versions of the algorithm, each adding a layer of complexity. Source code for all three energy models can be downloaded from http://bioinformatics.bc.edu/clotelab/.

The plan of this paper is as follows. Section “Results” discusses the degree distribution for move sets MS1 and MS2, obtained by exhaustive enumeration and by sampling low energy structures. Asymptotic network degree is discussed and the correlation is computed between the expected network degree, contact order, conformational entropy, and expected number of native contacts. In Section “Homopolymer Model A”, we derive the recursions for the expected number of neighbors for move set MS2, with respect to the *homopolymer* Model A. In the homopolymer model, introduced in [35], any two positions *i* < *j* can form a base pair, provided only that *j* − *i* > 1; i.e. in Definition 1, item (1) is removed, and item (2) is modified so that *θ* = 1. In this model, the partition function *Z* of a length *n* homopolymer is simply the number of well-balanced parenthesis expressions with dots, having length *n* and in which *j* − *i* > 1 whenever a left [resp. right] parenthesis occurs at position *i* [resp. *j*]. For this model, the probability *P*(*s*) of each structure *s* is equal to the uniform probability 1/*Z*. In Section “Uniform, non-homopolymer Model B”, we give the recursions for the non-homopolymer *uniform* Model B, in which every secondary structure has energy zero, but where a secondary structure of the RNA sequence **a** = *a*_1_, …, *a*_*n*_ must satisfy all four properties of Definition 1. In this case, the probability *P*(*s*) of structure *s* is defined by *P*(*s*) = exp(−*E*(*s*)/*RT*)/*Z* where *R* = 0.00198717 kcal/mol, *T* is absolute temperature, and the partition function is *Z* = ∑_*s*_ exp(−*E*(*s*)/*RT*). However, since *E*(*s*) = 0 for each structure *s*, the partition function *Z* is simply the number of secondary structures of **a**, and the probability *P*(*s*) is equal to the uniform probability *P*(*s*) = 1/*Z*. In Section “Model C with Turner energy parameters”, we give the the recursions for the full Model C, with respect to the Turner energy model [36] which includes base stacking free energies and free energies for hairpins, bulges, internal loops and multiloops. The partition function *Z* = ∑_*s*_ exp(−*E*(*s*)/*RT*) can be computed by the McCaskill algorithm [39], and the probability of structure *s* is the usual Boltzmann probability *P*(*s*) = exp(−*E*(*s*)/*RT*)/*Z*.

## Materials and Methods

Let **a** = *a*_1_, …, *a*_*n*_ be an arbitrary but fixed RNA sequence. For any 1 ≤ *i* ≤ *j* ≤ *n*, let *a*[*i, j*] denote the subsequence *a*_*i*_, …, *a*_*j*_, and let SS[i,j] denote the set of secondary structures of *a*[*i, j*]. For s∈SS[i,j], let *BF*(*s*) denote the Boltzmann factor exp(−*E*(*s*)/*RT*) of *s*, and define $Q_{i,j}=\sum_{s\in \text{SS}[i,j]}BF(s)\cdot N(s)$, where *N*(*s*) is the number of secondary structures *t* of *a*[*i, j*] obtained from the structure *s* by the addition, deletion or shift of a base pair. The partition function for *a*[*i, j*] is defined by $Z_{i,j}=\sum_{s\in \text{SS}[i,j]}BF(s)$. It follows that the expected number of neighbors (network degree) is $\frac{Q_{1,n}}{Z_{1,n}}$. For clarity of exposition, in the following subsections, we describe recursions to compute *Q*_*i,j*_ and *Z*_*i*,*j*_ for three energy models for RNA secondary structures, each model a refinement of the previous model.

### Homopolymer Model A

In this section, we derive the recursions for *Q*_1,*n*_ and *Z*_1,*n*_ for the homopolymer model, in which any two positions 1 ≤ *i* < *j* ≤ *n* can form a base pair, provided only that *i* + 1 < *j*. For the homopolymer model, there is no RNA sequence **a** = *a*_1_, …, *a*_*n*_, but rather only the interval [1, *n*] = {1, …, *n*}. Thus we speak of a structure on [*i, j*], rather than on *a*[*i, j*]. The energy of each structure in the homopolymer model is zero, so the probability of each structure *s* on [*i, j*] equals one divided by the number of structures on [*i, j*]. Moreover, there is no need to compute the doubly-indexed values *Q*_*i*,*j*_ and *Z*_*i*,*j*_, since the values depend only on the size *j* − *i* + 1 of the sequence [*i, j*]; i.e. if *j* − *i* = *j*′ − *i*′, then *Q*_*i*,*j*_ = *Q*_*i*′,*j*′_ and *Z*_*i*,*j*_ = *Z*_*i*′,*j*′_. Thus it is notationally simpler to define *Q*_*n*_ [resp. *Z*_*n*_] in place of *Q*_1,*n*_ [resp. *Z*_1,*n*_], and similarly for all other auxilliary functions.

For 0 ≤ *n*, define *Q*_*n*_ to be the sum, taken over all structures *s* of [1, *n*], of the number of base pair additions, removals or shifts of a base pair of *s*. Formally, we have

$$\begin{array}{ccc} Q_{n} & = & \sum_{s\in SS[1,n]}\sum_{(x,y)\in s}\sum_{k=1}^{n-2}\sum_{\ell =k+2}^{n}I\left[((x,y)\to (k,\ell ))\in \text{MS2},\left(s\setminus \left\{\left(x,y\right)\right\}\right)\cup \left\{\left(k,\ell \right)\right\}\;\text{is}\;\text{a}\;\text{valid}\;\text{str}\right] \end{array}$$ (1)

where *I* denotes the indicator function, and “(*x, y*) → (*k*, ℓ)” denotes the move which consists of replacing base pair (*x, y*) by base pair (*k*, ℓ). As well, let *Z*_*n*_ denote the total number of homopolymer structures on [1, *n*] with *θ* = 1. Recursions for *Z*_*n*_ are well-known [35], but for completeness given in Eq (2) below.

#### Auxilliary functions *f*(*n, x*) and *g*(*n, x*)

Recall that here we take *θ* = 1 for simplicity of exposition of the ideas. Let *Z*_*n*_ denote the total number of structures on the homopolymer of length *n*. Since any two positions *i, j* can base-pair, as long as *j* − *i* > *θ* = 1, we have

$$\begin{array}{ccc} Z_{n} & = & \left\{ \begin{array}{cc} 1 & \text{if}\;0\leq n\leq 2\\ Z_{n-1}+\sum_{r=1}^{n-2}Z_{r}\cdot Z_{n-r-2} & \text{otherwise.} \end{array}\right. \end{array}$$ (2)

The term *Z*_*n* − 1_ counts all structures *s* on [1, *n*] in which *n* is unpaired in *s*, while the term *Z*_*r*_ ⋅ *Z*_*n* − *r* − 2_ counts all structures *s* on [1, *n*] that contain the base pair (*r* + 1, *n*).

Define *f*(*n, x*) to be the number of secondary structures *s* for a length *n* homopolymer, such that *s* has *x* visible positions. Now for 0 ≤ *n* and 0 ≤ *x* ≤ *n*, define *f* by

$$\begin{array}{ccc} f(n,x) & = & \left\{ \begin{array}{cc} 1 & \text{if}\;n=0\text{,}\;x=0\\ 0 & \text{if}\;n=0\text{,}\;x>0\\ Z_{n-2}+\sum_{r=1}^{n-3}f\left(r,0\right)\cdot Z_{n-r-2} & \text{if}\;n>0\text{,}\;x=0\\ f\left(n-1,x-1\right)+\sum_{r=1}^{n-3}f\left(r,x\right)\cdot Z_{n-r-2} & \text{if}\;n>0\text{,}\;x>0 \end{array}\right. \end{array}$$ (3)

The computation of *f*(*n, x*) uses dynamic programming and proceeds by double induction, i.e. for *n* fixed, induction is performed on *x*. The term *Z*_*n* − 2_ arises from structures *s* on [1, *n*] that contain the base pair (1, *n*); the term *f*(*n* − 1, *x* − 1) is the contribution from structures *s* on [1, *n*] in which *n* is unpaired; the term *f*(*r, x*) ⋅ *Z*_*n* − *r* − 2_ accounts for all structures *s* on [1, *n*] that contain the base pair (*r* + 1, *n*).

Define *g*(*n, x*) to be the number of secondary structures *s* for the length *n* homopolymer, such that *s* has *x* visible positions in the interval [1, *n* − *θ* − 1] = [1, *n* − 2], and position *n* is unpaired in *s*.

$$\begin{array}{ccc} g(n,x) & = & \left\{ \begin{array}{cc} 0 & \text{if}\;0\leq n\leq 2\text{,}\;\text{for}\;\text{all}\;x\\ f\left(n-2,0\right)+Z_{n-3}+\sum_{r=1}^{n-4}f\left(r,0\right)\cdot Z_{n-r-3} & \text{if}\;n>2\text{,}\;x=0\\ f\left(n-2,x\right)+\sum_{r=1}^{n-4}f\left(r,x\right)\cdot Z_{n-r-3} & \text{if}\;n>2\text{,}\;x>0 \end{array}\right. \end{array}$$ (4)

The term *f*(*n* − 2, *x*) accounts for all structures *s* on [1, *n*] in which *n* − 1, *n* are unpaired. The term *Z*_*n* − 3_ arises in the case *n* > 2, *x* = 0 for structures *s* on [1, *n*] that contain the base pair (1, *n* − 1). Finally, the term *f*(*r, x*) ⋅ *Z*_*n* − *r* − 3_ arises from structures *s* on [1, *n*] that contain the base pair (*r* + 1, *n* − 1). In all cases, the structures considered are unpaired at position *n*, and have exactly *x* visible positions in the interval [1, *n* − 2].

#### Auxilliary function *E*_*n*_

For 1 ≤ *n*, define the function *E*_*n*_ to be the number of *external base pairs* in all homopolymer structures on [1, *n*]; formally, we have

$$\begin{array}{ccc} E_{n} & = & \sum_{s\in SS[1,n]}\sum_{\left(x,y\right)}I\left[\left(x,y\right)\;\text{is}\;\text{an}\;\text{external}\;\text{base}\;\text{pair}\;\text{in}\;s\right] \end{array}$$ (5)

Recalling that *Z*_*n*_ denotes the number of structures on [1, *n*], we define *Z*_0_ = 1, *E*_0_ = 1, and *E*_*n*_ = 0 for 1 ≤ *n* ≤ 2 = *θ* + 1. Note that for 1 ≤ *n* ≤ 2, it must be that *E*_*n*_ = 0, since the empty structure is the only possible structure on [1, *n*] in this case. For larger values of *n*, note that

$$\begin{array}{ccc} E_{n} & = & \sum_{s\in SS[1,n]}\sum_{1\leq x<y\leq n}I\left[\left(x,y\right)\;\text{is}\;\text{external}\;\text{base}\;\text{pair}\;\text{in}\;s\right]\\ & = & \sum_{s\in SS[1,n-1]}\sum_{1\leq x<y\leq n-1}I\left[\left(x,y\right)\;\text{is}\;\text{external}\;\text{base}\;\text{pair}\;\text{in}\;s\right]+\\ & & \sum_{k=1}^{n-\theta -1}\sum_{s_{1}\in SS\left[1,k-1\right]}\sum_{s_{2}\in SS\left[k,n\right]}\sum_{1\leq x<y\leq n}I\left[\left(x,y\right)\;\text{external}\;\text{in}\;s=s_{1}s_{2}\;\text{and}\;\left(k,n\right)\in s_{2}\right] \end{array}$$ (6)

$$\begin{array}{ccc} & = & E_{n-1}+\sum_{k=1}^{n-\theta -1}\sum_{s_{1}\in SS\left[1,k-1\right]}\sum_{s_{2}\in SS\left[k,n\right]}\sum_{1\leq x<y\leq k-1}I\left[\left(x,y\right)\;\text{external}\;\text{in}\;s_{1}\right]\cdot I\left[\left(k,n\right)\in s_{2}\right]+\\ & & \sum_{k=1}^{n-\theta -1}\sum_{s_{1}\in SS\left[1,k-1\right]}\sum_{s_{2}\in SS\left[k,n\right]}I\left[\left(k,n\right)\;\text{external}\;\text{in}\;s_{2}\right] \end{array}$$ (7)

$$\begin{array}{ccc} & = & E_{n-1}+\sum_{k=1}^{n-\theta -1}\sum_{s_{1}\in SS\left[1,k-1\right]}\sum_{1\leq x<y\leq k-1}I\left[\left(x,y\right)\;\text{external}\;\text{in}\;s_{1}\right](\sum_{s_{2}\in SS\left[k,n\right]}I\left[\left(k,n\right)\in s_{2}\right])+\\ & & \sum_{k=1}^{n-\theta -1}\sum_{s_{1}\in SS\left[1,k-1\right]}\sum_{s_{2}\in SS\left[k,n\right]}I\left[\left(k,n\right)\;\text{external}\;\text{in}\;s_{2}\right]\\ & = & E_{n-1}+\sum_{k=1}^{n-\theta -1}E_{k-1}\cdot Z_{n-k-1}+\sum_{k=1}^{n-\theta -1}Z_{k-1}\cdot Z_{n-k-1} \end{array}$$ (8)

Note that the rightmost term in the last line arises from the contribution of 1 for base pair (*k, n*). In summary, we have shown that

$$\begin{array}{ccc} E_{n} & = & \left\{ \begin{array}{cc} 1 & \text{if}\;n=0\\ 0 & \text{if}\;1\leq n\leq 2\\ E_{n-1}+\sum_{k=1}^{n-\theta -1}(E_{k-1}+Z_{k-1})\cdot Z_{n-k-1} & \text{otherwise.} \end{array}\right. \end{array}$$ (9)

#### Main function *Q*_*n*_

For clarity in the derivation of *Q*_*n*_, we start by explicitly listing the moves in move set MS2. Let *x, x*′, *y, y*′ denote distinct positions all belonging to the interval [1, *n*]. The structure *t* can be obtained from structure *s* by a move from MS2, if *t* is a valid secondary structure and can be obtained from *s* by applying a move of the form 1–6.

1. Addition of a base pair (*x, y*) to *s*.
2. Removal of a base pair (*x, y*) from *s*.
3. Shift of a base pair (*x, y*) in *s* to (*x, y*′) in *t*.
4. Shift of a base pair (*x, y*) in *s* to (*y*′, *x*) in *t*.
5. Shift of a base pair (*x, y*) in *s* to (*x*′, *y*) in *t*.
6. Shift of a base pair (*x, y*) in *s* to (*y, x*′) in *t*.

**Fig 8:**
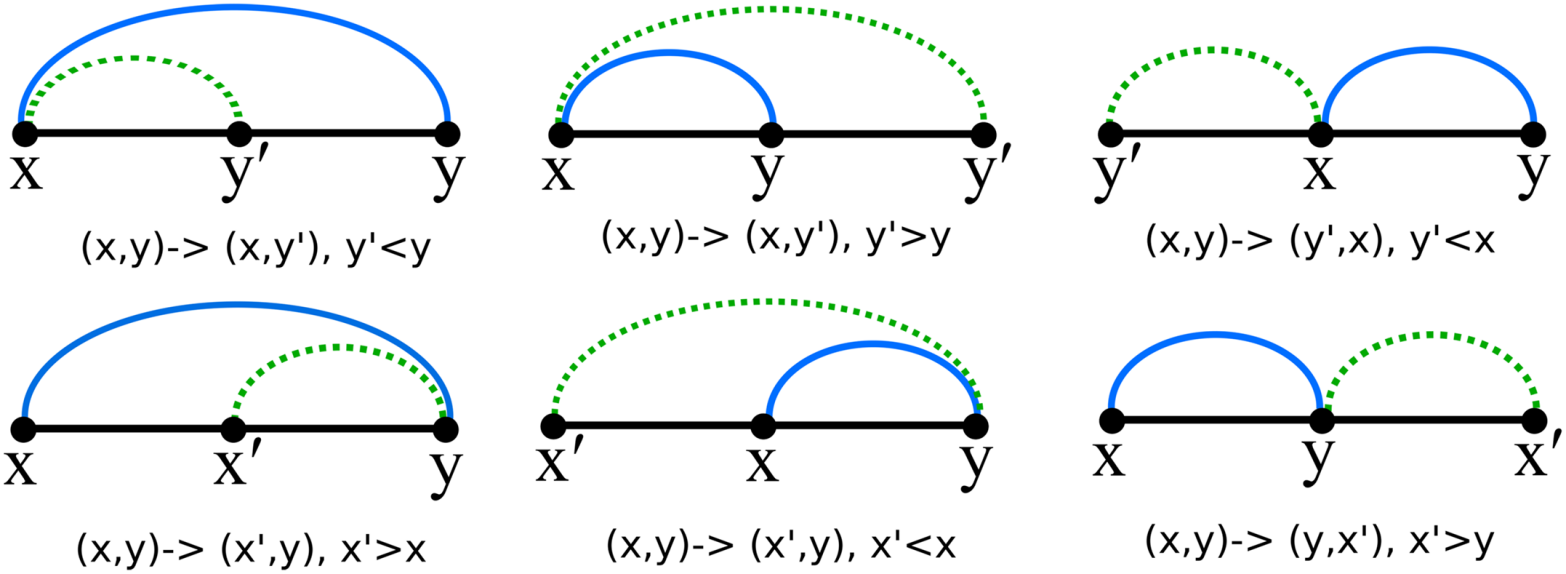
Illustration of shift moves defined in Sections “Main function *Q*_*n*_” and “Recursion for function *Q*_*i,j*_”.

The shift moves 3–6 are depicted in Fig 8.

Let $Q_{n}=\sum_{s\in \text{SS}[1,n]}N(s)$, where *N*(*s*) is the number of structures *t* that can be obtained from *s* by applying a move from move set MS2. Define *Q*_0_ = 1, and *Q*_1_ = *Q*_2_ = 0, *Z*_−1_ = 0, *Z*_0_ = *Z*_1_ = *Z*_2_ = 1. For the inductive case where *n* > 2, initialize *Q*_*n*_ = 0 and then add the contributions from below.

Case 1(a): In this case, we consider the contribution from s∈SS[1,n], in which the last position *n* is unpaired, and *t* is obtained from *s* by a move from MS2 involving *x, y, x*′, *y*′ ∈ [1, *n* − 1].

Notice that in shifts of type 3, 4 the original position *x* is retained, while in shifts of type 5, 6 the original position *y* is retained, for distinct *x, x*′, *y* in the interval [1, *n* − 1]. Also, notice that shifts of base pairs involving the last position *n* are not considered in Case 1(a) – such shifts will later be treated in cases 1(c), 2(b) and 2(c). The contribution in this case is given by

$$\begin{array}{ccc} Q_{n}^{\left(1a\right)} & = & Q_{n-1}. \end{array}$$ (10)

The term *Q*_*n*−1_ arises from neighbors *t* of *s* in which the last position *n* is unpaired, and the base pair (*x, y*) is added/removed/shifted in *s*.

Case 1(b): In this case, we consider the contribution from s∈SS[1,n], in which the last position *n* is unpaired, and *t* is obtained from *s* by adding the base pair (*k, n*) for some 1 ≤ *k* ≤ *n* − *θ* − 1. The contribution in this case is given by

$$\begin{array}{ccc} Q_{n}^{\left(1b\right)} & = & \sum_{k=1}^{n-\theta -1}Z_{k-1}\cdot Z_{n-k-1}. \end{array}$$ (11)

Case 1(c): In this case, we consider the contribution from s∈SS[1,n], in which the last position *n* is unpaired, and *t* is obtained from *s* by shifting the base pair (*x, y*) to (*x, n*), or by shifting the base pair (*x, y*) to (*y, n*), for distinct *x, y* in the interval [1, *n* − 1]. These shifts are treated separately.

Case 1(c)(i): Consider a shift of the form (*x, y*) to (*x, n*), for *y* < *n*. The function *E*_*n*−1_ counts the number of external base pairs (*x, y*) where *y* ≤ *n* − 1, for all structures on [1, *n* − 1]. For any such (*x, y*), it is possible to shift the base pair (*x, y*) to (*x, n*), and so the contribution is

$$\begin{array}{c} E_{n-1} \end{array}$$ (12)

Case 1(c)(ii): Consider a shift of the form (*x, y*) to (*y, n*), for *y* < *n* − 1. The function *E*_*n*−2_ counts the sum over all structures on [1, *n* − 2] of the number of external base pairs (*x, y*) with *y* ≤ *n* − 2. Since *k* ≤ *n* − 2 and *θ* = 1, and *n* is unpaired, it is possible to shift the base pair (*x, y*) to (*y, n*) and vice versa. So far, we have not considered structures *s* on [1, *n* − 1] in which *n* − 1 is base-paired. For a structure *s* on [1, *n* − 1] that contains base pair (*r* + 1, *n* − 1), there are *Z*_*n*−*r*−3_ many structures *s*_2_ on [*r* + 2, *n* − 2]; moreover, for any external base pair (*x, y*) in a structure *s*_1_ on [1, *r*], we can shift the base pair (*x, y*) to (*y, n*). This explains the presence of the term $\sum_{r=1}^{n-4}E_{r}\cdot Z_{n-r-3}$. Thus the contribution is

$$\begin{array}{c} E_{n-2}+\sum_{r=1}^{n-4}E_{r}\cdot Z_{n-r-3}. \end{array}$$ (13)

In conclusion,

$$\begin{array}{ccc} Q_{n}^{\left(1c\right)} & = & E_{n-1}+E_{n-2}+\sum_{r=1}^{n-4}E_{r}\cdot Z_{n-r-3}. \end{array}$$ (14)

Case 2(a): The contribution from s∈SS[1,n], in which the last position *n* is base-paired, where neighbor *t* is obtained from *s* by removal of that last base pair (*k, n*), is given by

$$\begin{array}{ccc} Q_{n}^{\left(2a\right)} & = & \sum_{k=1}^{n-\theta -1}Z_{k-1}\cdot Z_{n-k-1} \end{array}$$ (15)

Note that Case 2(a) is dual to Case 1(b).

Case 2(b): In this case, we consider the contribution from s∈SS[1,n], in which the last position *n* is base-paired, where neighbor *t* is obtained from structure *s* by a shift of the last base pair (*k, n*) to (*k*′, *n*) for some *k*′ ≠ *k* that is visible in structure *s* − {(*k, n*)}. Note that if we were to remove base pair (*k, n*) from *s*, then the last position of *s* − {(*k, n*)} must be unpaired, and the position *n* − 1 may or may not be base paired. Recall that *g*(*n, x*) is the sum over all structures *s* on [1, *n*], that contain *x* visible positions in the interval [1, *n* − 2], and in which position *n* is unpaired. If we *choose* a first position *k* out of the *x* visible positions, and subsequently a second distinct position *k*′ out of the remaining *x* − 1 visible positions, then we properly count the contribution from structures *s* containing (*k, n*) which can be transformed to a structure *t* by the shift (*k*′, *n*).

The contribution in this case is

$$\begin{array}{ccc} Q_{n}^{\left(2b\right)} & = & \sum_{x=2}^{n-\theta -1}x\left(x-1\right)\cdot g\left(n,x\right). \end{array}$$ (16)

since we have *x* choices for value *k* and then (*x* − 1) choices for *k*′, both selected from the *x* visible positions of the structure.

Case 2(c): In this case, we consider the contribution from s∈SS[1,n], in which the last position *n* is base-paired, where neighbor *t* is obtained from structure *s* by a shift of base pair (*k, n*) to (*k, k*′), or a shift of the last base pair (*k, n*) to (*k*′, *k*), for some *k* ≠ *k*′ that is visible in structure *s* − {(*k, n*)}. These shifts are treated separately.

Case 2(c)(i): Consider a shift of the form (*k, n*) to (*k, k*′), for *k*′ < *n*. The function *E*_*n*−1_ counts the sum over all structures on [1, *n* − 1] of the number of external base pairs (*k, k*′) with *k*′ ≤ *n* − 1. For any such (*k, k*′), it is possible to apply the shift (*k, n*), and vice versa. Thus Case 2(c)(i) case is dual to Case 1(c)(i) and the contribution is clearly

$$\begin{array}{c} E_{n-1} \end{array}$$ (17)

Case 2(c)(ii): Consider a shift of the form (*k, n*) to (*k*′, *k*), for *k*′ < *k* − 1. The function *E*_*n*−2_ counts the sum over all structures on [1, *n* − 2] of the number of external base pairs (*k*′, *k*) with *k* ≤ *n* − 2. Since *k* ≤ *n* − 2 and *θ* = 1, and *n* is unpaired, it is possible to shift the base pair (*k*′, *k*) to (*k, n*) and vice versa. By duality to Case 1(c)(ii), we have the additional contribution of $\sum_{r=1}^{n-4}E_{r}\cdot Z_{n-r-3}$ to account for shifting the base pair (*y, n*) to an external base pair (*x, y*) in a structure *s*_1_ on [1, *r*], in the case that *n* − 1 is base-paired. Thus Case 2(c)(ii) case is dual to Case 1(c)(ii) and the contribution is clearly

$$\begin{array}{c} E_{n-2}+\sum_{r=1}^{n-4}E_{r}\cdot Z_{n-r-3}. \end{array}$$ (18)

In conclusion,

$$\begin{array}{ccc} Q_{n}^{\left(2c\right)} & = & E_{n-1}+E_{n-2}+\sum_{r=1}^{n-4}E_{r}\cdot Z_{n-r-3}. \end{array}$$ (19)

Case 2(d): In this case, we consider the contribution from s∈SS[1,n], in which the last position *n* is base-paired with base pair (*k, n*), where neighbor *t* is obtained from a shift or addition/deletion of a base pair in the left portion [1, *k* − 1] or right portion [*k* + 1, *n* − 1], so that *t* retains the base pair (*k, n*). In this case, the contribution is

$$\begin{array}{ccc} Q_{n}^{\left(2d\right)} & = & \sum_{k=1}^{n-\theta -1}(Z_{k-1}\cdot Q_{n-k-1}+Q_{k-1}\cdot Z_{n-k-1}). \end{array}$$ (20)

The first term arises from the addition/removal/shift of a base pair (*x, y*), where *k* + 1 ≤ *x* < *y* ≤ *n* − 1, and the second term arises from the addition/removal/shift of a base pair (*x, y*), where 1 ≤ *x* < *y* ≤ *k*−1.

Putting together all contributions from Case 1(a) through Case 2(d), we have

$$\begin{array}{ccc} Q_{n} & = & Q^{\left(1a\right)}+Q^{\left(1b\right)}+Q^{\left(1c\right)}+Q^{\left(2a\right)}+Q^{\left(2b\right)}+Q^{\left(2c\right)}+Q^{\left(2d\right)}\\ & = & Q_{n-1}+2\sum_{k=1}^{n-\theta -1}Z_{k-1}\cdot Z_{n-k-1}+2(E_{n-1}+E_{n-2}+\sum_{r=1}^{n-4}E_{r}\cdot Z_{n-r-3})+\\ & & \sum_{x=2}^{n-\theta -1}x\left(x-1\right)\cdot g\left(n,x\right)+\sum_{k=1}^{n-\theta -1}(Z_{k-1}\cdot Q_{n-k-1}+Q_{k-1}\cdot Z_{n-k-1}) \end{array}$$ (21)

The functions *f, g* require the greatest space and time resources, and it is easily seen that the spece [resp. time] complexity for *Z* is *O*(*n*) [resp. *O*(*n* ^2^)], for *f* is *O*(*n* ^2^) [resp. *O*(*n* ^3^)], for *g* is *O*(*n* ^2^) [resp. *O*(*n* ^3^)], and that given arrays that contain the values of *f* and *g*, the additional space [resp. time] complexity for *E* and *Q* is *O*(*n*) [resp. *O*(*n* ^2^)]. It follows that the expected network degree in the homopolymer case Model A can be computed in quadratic space *O*(*n* ^2^) and cubic time *O*(*n* ^3^). We have implemented a dynamic programming algorithm for each of the functions *E, f, g, Q, Z* resulting in software for the expected network degree, with respect to homopolymer model. Our code has been cross-checked extensively with alternative brute-force methods, hence is reliable.

### Uniform, non-homopolymer Model B

In this section, we consider the uniform, non-homopolymer model B, in which secondary structures must satisfy Definition 1; i.e. compared with the notion of structure from the previous Section “Homopolymer Model A”, each base pair (*i, j*) of a secondary structure *s* of the RNA sequence **a** = *a*_1_, …, *a*_*n*_ must satisfy *j* − *i* > *θ* = 3, and *a*_*i*_, *a*_*j*_ must constitute a Watson-Crick or wobble pair. In model B, the energy of each structure is zero, so the partition function *Z* = *Z*_1,*n*_ is the total number of structures of **a**, and the probability *P*(*s*) of each structure *s* is 1/*Z*. For the recursions necessary to compute $Q_{i,j}=\sum_{s\in \text{SS}[i,j]}\;N(s)$, where *N*(*s*) denotes the number of neighbors of *s* under move set MS2, we need to define new functions *EL, ER, ER*′, *F, G*. There is a correspondence between functions *EL*_*i,j* − 1, *a*_*j*__ [resp. $ER_{i,j,a_{j}}^{\prime }$] { resp. *G*_*i,j,a*_*j*_, *x*_ } in the current section with the functions *E*_*n*−1_ [resp. $E_{n-2}+\sum_{r=1}^{n-r-\theta -1}E_{r}\cdot Z_{n-r-3}$] { resp. *g*(*n, x*) } from the previous Section “Homopolymer Model A”.

#### Critical definitions and recursions

For a given RNA sequence **a** = *a*_1_, …, *a*_*n*_, define the subsequence **a**[*i, j*] = *a*_*i*_, …, *a*_*j*_. Positions *i, j* can form a base pair, denoted by *bp*(*i, j*) = 1, if *a*_*i*_, *a*_*j*_ is either a Watson-Crick pair AU, UA, GC, or CG, or a wobble pair; otherwise *bp*(*i, j*) = 0. For *k* ∈ [1, *n*] and *c* ∈ {*A, C, G, U*}, we also write *bp*(*k, c*) = 1 to mean that *a*_*k*_, *c* constitute either a Watson-Crick or wobble base pair. A nucleotide position *k* ∈ [1, *n*] is said to be *visible* in the secondary structure *s*, if for every base pair (*i, j*) ∈ *s*, it is *not* the case that *i* ≤ *k* ≤ *j*. If we state that structure *s* has exactly *x* visible occurrences of a nucleotide in [*i, j* − *θ* − 1] that can base pair with *c*, then we mean that there are positions *i* ≤ *i*_1_ < *i*_2_ < ⋯ < *i*_*x*_ ≤ *j* − *θ* − 1 visible in *s*, such that *bp*(*i*_1_, *c*) = 1, …, *bp*(*i*_*x*_, *c*) = 1; moreover there are *no other* positions beyond *i*_1_, …, *i*_*x*_ with this property.

The base pair (*i, j*) ∈ *s* is said to be an *external* base pair of the secondary structure *s*, if there is no distinct base pair (*i*′, *j*′) ∈ *s* with the property that *i*′ ≤ *i* < *j* ≤ *j*′. In formulas, for brevity, we write that ‘(*i, j*) is external in *s*’, to mean that (*i, j*) is an external base pair of *s*. Let SS[i,j] denote the set of all secondary structures of the subword **a**[*i, j*]. Recall that the indicator function *I*[*P*] is equal to 1 if relation *P* is true, and 0 otherwise. For 1 ≤ *i* ≤ *j* ≤ *n, c* ∈ {*A, C, G, U*}, and *x* ∈ [0, *n*], and *c* ∈ {*A, C, G, U*}, define the functions *EL*_*i,j,c*_, *ER*_*i,j,c*_, $ER_{i,j,c}^{\prime }$, *F*_*i,j,c,x*_, *G*(*i,j,c,x*) as follows.

$$\begin{array}{ccc} EL_{i,j,c} & = & \sum_{s\in SS[i,j]}\sum_{\left(x,y\right)}I[\left(x,y\right)\;\text{is}\;\text{external}\;\text{bp}\;\text{in}\;s\text{,}\;bp(x,c)=1] \end{array}$$ (22)

$$\begin{array}{ccc} ER_{i,j,c} & = & \sum_{s\in SS[i,j]}\sum_{\left(x,y\right)}I[\left(x,y\right)\;\text{is}\;\text{external}\;\text{bp}\;\text{in}\;s\text{,}\;bp(y,c)=1] \end{array}$$ (23)

$$\begin{array}{ccc} ER_{i,j,c}^{\prime } & = & \sum_{s\in SS[i,j]}\sum_{\left(x,y\right)}I[(x,y)\in s\;\text{is}\;\text{ext.}\;\text{bp}\;\text{in}\;s\text{,}\;bp(y,c)=1\text{,}\;y\leq j-\theta -1\text{,}\;j\;\text{unpaired}\;\text{in}\;s\;] \end{array}$$ (24)

$$\begin{array}{ccc} F_{i,j,c,x} & = & \sum_{s\in SS[i,j]}I[s\;\text{has}\;\text{exactly}\;x\;\text{visible}\;\text{occurrences}\;\text{of}\;\text{a}\;\text{nucleotide}\;\text{that}\;\text{can}\;\text{pair}\;\text{with}\;c] \end{array}$$ (25)

$$\begin{array}{ccc} G_{i,j,c,x} & = & \sum_{s\in SS[i,j]}I[s\;\text{has}\;\text{exactly}\;x\;\text{visible}\;\text{occurrences}\;\text{of}\;\text{a}\;\text{nucleotide}\;\text{in}\;\left[1,j-\theta -1\right]\\ & & \text{that}\;\text{can}\;\text{pair}\;\text{with}\;c\text{,}\;\text{and}\;j\;\text{unpaired}\;\text{in}\;s] \end{array}$$ (26)

**Fig 9:**
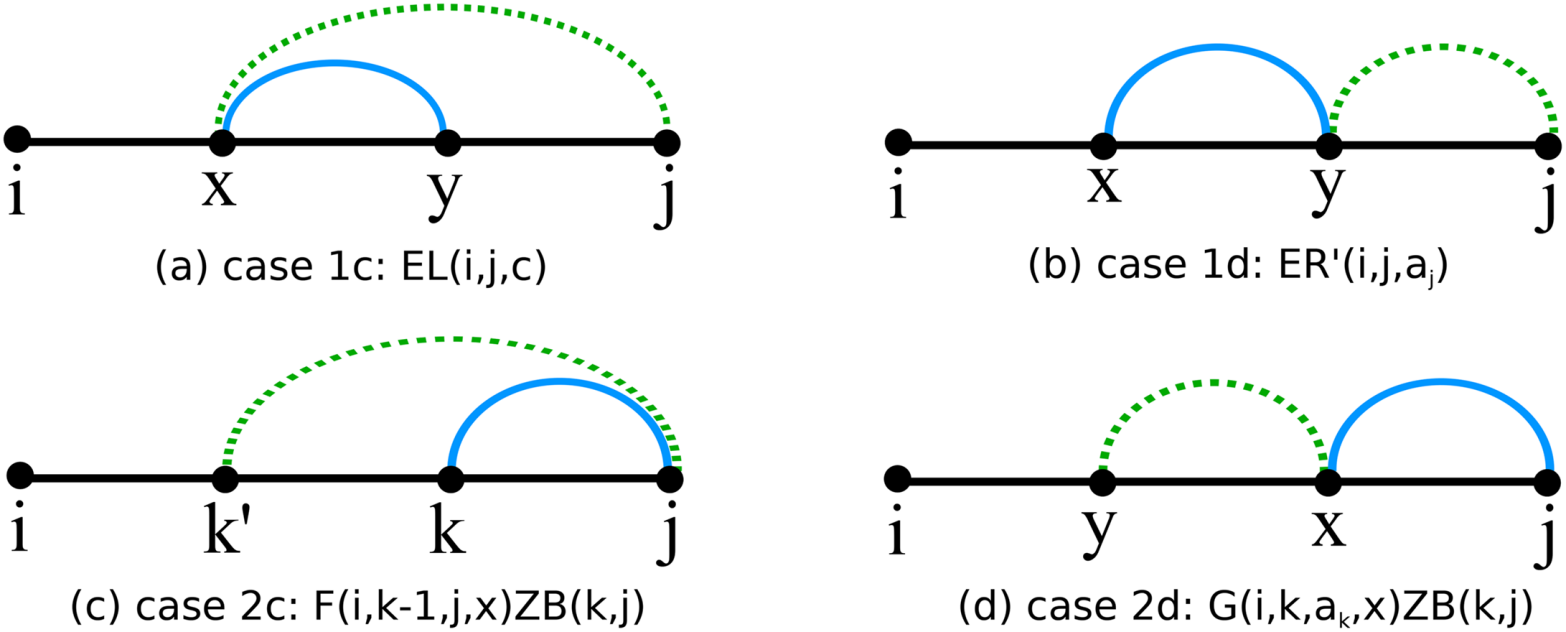
Illustration of cases 1c, 1d, 2c, 2d from Section “Recursion for function *Q*_*i,j*_”.

The two differences between the homopolymer Model A and the current Model B are: (1) in Model B, if (*k, j*) is a base pair, then the nucleotides at positions *k, j* must be one of AU, UA, GC, CG, GU, UG, (2) in Model B, *θ* = 3, so if (*k, j*) is a base pair, then *j* ≥ *i* + *θ* + 1 = *i* + 4. Both of these issues substantially complicate the treatment, so instead of the function *E*_*n*_ with one argument, we have three functions, *EL*_*i,j,c*_, *ER*_*i,j,c*_, $ER_{i,j,c}^{\prime }$, each having three arguments. The arguments *i, j* designate the left and right endpoints of the interval [*i, j*], and the functions are defined by induction on increasing values of the difference *j* − *i*. The argument *c* contains the value A, C, G, U for the nucleotide at position *j*; this allows one to test whether the nucleotide at position *k* ∈ [*i, j* − *θ* − 1] can form a base pair with the nucleotide at position *j*. Thus *EL*_*i,j,c*_ is the sum, taken over all structures on [*i, j*], of the number of external base pairs (*x, y*) where we can alternatively form the base pair (*x, j*) as depicted in panel (a) of Fig 9. As well, $ER_{i,j,c}^{\prime }$ is the sum, taken over all structures on [*i, j*], of the number of external base pairs (*x, y*) where we can alternatively form the base pair (*y, j*) as depicted in panel (b) of Fig 9. The function *ER*_*i,j,c*_ is first defined, since this simplifies the recursion for $ER_{i,j,c}^{\prime }$. The function *G*_*i,j,c,x*_ has a fourth parameter *x*, for which *G*_*i,j,c,x*_ counts the number of structures on [*i, j*] having *exactly* *x* visible positions (external to all base pairs) in the interval [*i, j* − *θ* − 1] = [*i, j* − 4] of a nucleotide that can form a base pair with nucleotide *c*, as depicted in panel (d) of Fig 9. It will follow that for structures having exactly *x* such visible positions that can form a base pair with position *j*, there are $\left(\begin{array}{c} x\\ 2 \end{array}\right)=x\cdot (x-1)/2$ many pairs *k*′, *k* where a shift of the form (*k, j*) → (*k*′, *j*). The function *F*_*i,j,c,x*_ is introduced to simplify the recursions for *G*, where *F*_*i,j,c,x*_ counts the number of structures on [*i, j*] having *exactly* *x* visible occurrences of a nucleotide that can form a base pair with *c*. With this introduction, we give the formal definitions.

#### Definition of *EL*

For 1 ≤ *i* ≤ *j* ≤ *n* and *c* ∈ {*A, C, G, U*}, we define *EL*_*i,j,c*_ by induction on *j* − *i*.

Base Case: If *j* − *i* ≤ *θ*, define *EL*_*i,j,c*_ = 0.

Inductive Case: If *j* − *i* > *θ*, define *EL*_*i,j,c*_ as the sum of the following

$$\begin{array}{ccc} EL_{i,j,c} & = & EL_{i,j-1,c}+bp\left(i,j\right)\cdot bp\left(i,c\right)\cdot Z_{i+1,j-1}+\sum_{k=i+1}^{j}bp\left(k,j\right)\cdot EL_{i,k-1,c}\cdot Z_{k+1,j-1}+\\ & & \sum_{k=i+1}^{j}bp\left(k,j\right)\cdot bp\left(k,c\right)\cdot Z_{i,k-1}\cdot Z_{k+1,j-1} \end{array}$$ (27)

#### Definition of *ER*

For 1 ≤ *i* ≤ *j* ≤ *n* and *c* ∈ {*A, C, G, U*}, we define *ER*_*i,j,c*_ by induction on *j* − *i*.

Base Case: If *j* − *i* ≤ *θ*, define *ER*_*i,j,c*_ = 0.

Inductive Case: If *j* − *i* > *θ*, define *ER*_*i,j,c*_ as the sum of the following

$$\begin{array}{ccc} ER_{i,j,c} & = & ER_{i,j-1,c}+bp\left(i,j\right)\cdot bp\left(j,c\right)\cdot Z_{i+1,j-1}+\sum_{k=i+1}^{j}bp\left(k,j\right)\cdot ER_{i,k-1,c}\cdot Z_{k+1,j-1}+\\ & & \sum_{k=i+1}^{j}bp\left(k,j\right)\cdot bp\left(j,c\right)\cdot Z_{i,k-1}\cdot Z_{k+1,j-1} \end{array}$$ (28)

#### Definition of *ER*′

For 1 ≤ *i* ≤ *j* ≤ *n* and *c* ∈ {*A, C, G, U*}, we define $ER_{i,j,c}^{\prime }$ by induction on *j* − *i*.

Base Case: If *j* − *i* ≤ *θ*, define $ER_{i,j,c}^{\prime }=0$.

Inductive Case: If *j* − *i* > *θ*, define $ER_{i,j,c}^{\prime }$ as the sum of the following

$$\begin{array}{ccc} ER_{i,j,c}^{\prime } & = & ER_{i,j-\theta -1,c}+\\ & & \sum_{u=1}^{3}\sum_{k=i+1}^{j-\theta -1+u-\theta -1}bp\left(k,j-\theta -1+u\right)\cdot I\left[j-\theta -1+u-k>\theta \right]\cdot ER_{i,k-1,c}\cdot Z_{k+1,j-\theta -1+u-1} \end{array}$$ (29)

Note that the first term to the right of the equality sign in the previous equation is *ER*_*i,j*−*θ* − 1, *c*_ and *not* $ER_{i,j-\theta -1,c}^{\prime }$.

#### Definition of *F*

For 1 ≤ *i* ≤ *j* ≤ *n, c* ∈ {*A, C, G, U*} and *x* ∈ [0, *n*], we define *F*_*i,j,c,x*_ by induction on *j* − *i*. For *j* − *i* < 0, *c* ∈ {*A, C, G, U*}, and 0 ≤ *x* ≤ *j* − *i* + 1, define *F*_*i,j,c,x*_ = 0.

Base Case *i* = *j*: For *c* ∈ {*A, C, G, U*}, define *F*_*i,i,c,bp*(*i, c*)_; i.e.

$$\begin{array}{ccc} F_{i,i,c,0} & = & \left\{ \begin{array}{cc} 1 & \text{if}\;bp(i,c)=0\\ 0 & \text{else} \end{array}\right. \end{array}$$ (30)

and

$$\begin{array}{ccc} F_{i,i,c,1} & = & \left\{ \begin{array}{cc} 1 & \text{if}\;bp(i,c)=1\\ 0 & \text{else} \end{array}\right. \end{array}$$ (31)

Base Case *i* < *j* ≤ *i*+*θ*: For *i* < *j* ≤ *i* + *θ*, and *x* ∈ [0, *j* − *i* + 1], define by double induction on *j* − *i* and *x*

$$\begin{array}{ccc} F_{i,j,c,x} & = & \left\{ \begin{array}{cc} F_{i,j-1,c,x-1} & \text{if}\;x>0\;\text{and}\;bp(j,c)=1\\ F_{i,j-1,c,x} & \text{if}\;bp(j,c)=0 \end{array}\right. \end{array}$$ (32)

Inductive Case *j* > *i*+*θ*: For *j* > *i*+*θ*, and *x* ∈ [0, *n*], we define *F* by double induction on *j* − *i* and *x*, where we separate the case that *x* = 0 and *x* > 0.

Subcase *x* = 0:

$$\begin{array}{ccc} F_{i,j,c,0} & = & \left(1-bp\left(j,c\right)\right)\cdot F_{i,j-1,c,0}+bp\left(i,j\right)\cdot Z_{i+1,j-1}+\sum_{k=i+1}^{j-\theta -1}bp\left(k,j\right)\cdot F_{i,k-1,c,0}\cdot Z_{k+1,j-1} \end{array}$$ (33)

Subcase *x* > 0:

$$\begin{array}{ccc} F_{i,j,c,x} & = & bp\left(j,c\right)\cdot F_{i,j-1,c,x-1}+\sum_{k=i+1}^{j-\theta -1}bp\left(k,j\right)\cdot I\left[x\in \left[0,k-i\right]\right]\cdot F_{i,k-1,c,x}\cdot Z_{k+1,j-1} \end{array}$$ (34)

#### Definition of *G*

Recall that *G*_*i,j,c,x*_ is defined to be the number of structures s∈SS[i,j] having exactly *x* visible occurrences of a nucleotide in [*i, j* − *θ* − 1] that can base-pair with *c*, and *j* is unpaired in *s*. Initially define *G*_*i,j,c,x*_ = 0 for all *i, j, c, x*.

Base Case: For *i* ≤ *j* ≤ *i* + *θ*, and *c* ∈ {*A, C, G, U*}, define *G*_*i,j,c*, 0_ = 0.

Inductive Case: In this case, *j* > *i* + *θ*, and *c* ∈ {*A, C, G, U*}. We separately treat the subcases *x* = 0 and *x* > 0.

Subcase *x* = 0:

$$\begin{array}{ccc} G_{i,j,c,0} & = & F_{i,j-\theta -1,c,0}+\sum_{u=1}^{3}I\left[j-\theta -1+u-i>\theta \right]\cdot bp\left(i,j-\theta -1+u\right)\cdot Z_{i+1,j-\theta -1+u-1}+\\ & & \sum_{u=1}^{3}\sum_{k=i+1}^{j-\theta -1+u-\theta -1}I\left[j-\theta -1+u-k>\theta \right]\cdot bp\left(k,j-\theta -1+u\right)\cdot F_{i,k-1,c,0}\cdot Z_{k+1,j-\theta -1+u-1} \end{array}$$ (35)

Subcase *x* > 0:

$$\begin{array}{ccc} G_{i,j,c,x} & = & F_{i,j-\theta -1,c,x}+\\ & & \sum_{u=1}^{3}\sum_{k=i+1}^{j-\theta -1+u-\theta -1}I\left[j-\theta -1+u-k>\theta \right]\cdot bp\left(k,j-\theta -1+u\right)\cdot F_{i,k-1,c,x}\cdot Z_{k+1,j-\theta -1+u-1} \end{array}$$ (36)

#### Computing the total number of moves using MS1

For 1 ≤ *i* ≤ *j* ≤ *n*, define *Q*_*i,j*_ to be the sum, taken over all structures *s* of *a*_*i*_, …, *a*_*j*_, of the number of base pair additions or removals of a base pair to or from *s*. Formally, we have

$$\begin{array}{ccc} Q_{i,j} & = & \sum_{s\in SS[i,j]}\sum_{(x,y)\in s}\sum_{k=i}^{j-\theta -1}\sum_{\ell =k+\theta +1}^{j}I\left[((x,y)\to (k,\ell ))\in \text{MS1},(s\setminus \{(x,y)\})\cup \left\{\left(k,\ell \right)\right\}\;\text{valid}\;\text{str}\right] \end{array}$$ (37)

or equivalently

$$\begin{array}{ccc} Q_{i,j} & = & \sum_{s\in SS[i,j]}\sum_{t\in SS[i,j]}I\left[d_{\text{BP}}\left(s,t\right)=1\right] \end{array}$$ (38)

where *d*_BP_(*s, t*) denotes the base pair distance between structures *s, t*. Define *Q*_*i,j*_ by recursion on *j* − *i*, for 1 ≤ *i* ≤ *j* ≤ *n*.

Base Case: For *i* ≤ *j* ≤ *i* + *θ*, define *Q*_*i,j*_ = 0.

Inductive Case: For *j* > *i* + *θ*, define

$$\begin{array}{ccc} Q_{i,j} & = & Q_{i,j-1}+2\cdot (bp\left(i,j\right)\cdot Z_{i+1,j-1}+\sum_{k=i+1}^{j-\theta -1}bp\left(k,j\right)\cdot Z_{i,k-1}\cdot Z_{k+1,j-1})+\\ & & bp\left(i,j\right)\cdot Q_{i+1,j-1}+\sum_{k=i+1}^{j-\theta -1}bp\left(k,j\right)\cdot (Q_{i,k-1}\cdot Z_{k+1,j-1}+Z_{i,k-1}\cdot Q_{k+1,j-1}) \end{array}$$ (39)

#### Computing the total number of moves using MS2

For 1 ≤ *i* ≤ *j* ≤ *n*, define *Q*_*i,j*_ to be the sum, taken over all structures *s* of *a*_*i*_, …, *a*_*j*_, of the number of base pair additions, removals or shifts of a base pair of *s*. Formally, we have

$$\begin{array}{ccc} Q_{i,j} & = & \sum_{s\in SS[i,j]}\sum_{(x,y)\in s}\sum_{k=i}^{j-\theta -1}\sum_{\ell =k+\theta +1}^{j}I\left[((x,y)\to (k,\ell ))\in \text{MS2},(s\setminus \{(x,y)\})\cup \left\{\left(k,\ell \right)\right\}\;\text{is}\;\text{valid}\;\text{str}\right] \end{array}$$ (40)

Now define *Q*_*i,j*_ by recursion on *j* − *i*, for 1 ≤ *i* ≤ *j* ≤ *n*.

Base Case: For *i* ≤ *j* ≤ *i* + *θ*, define *Q*_*i,j*_ = 0.

Inductive Case: For *j* > *i* + *θ*, define

$$\begin{array}{ccc} Q_{i,j} & = & Q_{i,j-1}+2\cdot (bp\left(i,j\right)\cdot Z_{i+1,j-1}+\sum_{k=i+1}^{j-\theta -1}bp\left(k,j\right)\cdot Z_{i,k-1}\cdot Z_{k+1,j-1})+\\ & & 2\cdot (EL_{i,j-1,a_{j}}+ER_{i,j,a_{j}}^{\prime })+\sum_{x=2}^{j-i-\theta }x\cdot \left(x-1\right)\cdot G_{i,j,a_{j},x}+\\ & & bp\left(i,j\right)\cdot Q_{i+1,j-1}+\sum_{k=i+1}^{j-\theta -1}bp\left(k,j\right)\cdot (Q_{i,k-1}\cdot Z_{k+1,j-1}+Z_{i,k-1}\cdot Q_{k+1,j-1}) \end{array}$$ (41)

#### Computing the total number of moves using MS2\MS1

For 1 ≤ *i* ≤ *j* ≤ *n*, define *Q*_*i,j*_ to be the sum, taken over all structures *s* of *a*_*i*_, …, *a*_*j*_, of the number of shifts of a base pair of *s*. Formally, we have

$$\begin{array}{ccc} Q_{i,j} & = & \sum_{s\in SS[i,j]}\sum_{(x,y)\in s}\sum_{k=i}^{j-\theta -1}\sum_{\ell =k+\theta +1}^{j}\\ & & I[\left(x,y\right)\in s,((x,y)\to (k,\ell ))\in \left\{\text{MS2}\setminus \text{MS1}\right\},(s\setminus \{(x,y)\})\cup \left\{\left(k,\ell \right)\right\}\;\text{valid}\;\text{str}] \end{array}$$ (42)

Now define *Q*_*i,j*_ by recursion on *j* − *i*, for 1 ≤ *i* ≤ *j* ≤ *n*.

Base Case: For *i* ≤ *j* ≤ *i* + *θ*, define *Q*_*i,j*_ = 0.

Inductive Case: For *j* > *i* + *θ*, define

$$\begin{array}{ccc} Q_{i,j} & = & Q_{i,j-1}+2\cdot (EL_{i,j-1,a_{j}}+ER_{i,j,a_{j}}^{\prime })+\sum_{x=2}^{j-i-\theta }x\cdot \left(x-1\right)\cdot G_{i,j,a_{j},x}+\\ & & bp\left(i,j\right)\cdot Q_{i+1,j-1}+\sum_{k=i+1}^{j-\theta -1}bp\left(k,j\right)\cdot (Q_{i,k-1}\cdot Z_{k+1,j-1}+Z_{i,k-1}\cdot Q_{k+1,j-1}) \end{array}$$ (43)

We have implemented a dynamic programming algorithm for each of the functions *EL, ER, ER*′, *F, G, Q* and *Z*, resulting in software for the expected network degree, with respect to uniform probability for the move sets MS1, MS2, MS2\MS1. Analysis of space and time resources needed for the program can be determined in a manner similar to that described at the end of Subsection; however, there is an additional factor of *n* in both space and time requirements, so that the software runs in space *O*(*n* ^3^) and time *O*(*n* ^4^). During the algorithm development and implementation, we have extensively cross-checked with results obtained by exhaustive, brute force counting, thus ensuring correctness of our code.

### Model C with Turner energy parameters

Here we consider the Model C, for which secondary structures satisfy Definition 1 and such that *E*(*s*) indicates the Turner energy of *s*, which involves free energy parameters [36] for stacked base pairs, hairpins, bulges, internal loops and multiloops. For RNA sequence **a** = *a*_1_, …, *a*_*n*_, we present recursions in the following for *Z*_*i,j*_ and *Q*_*i,j*_, where

$$\begin{array}{ccc} N(s) & = & \sum_{t\in SS[i,j]}I\left[t\;\text{obtained}\;\text{from}\;s\;\text{by}\;\text{a}\;\text{move}\;\text{in}\;\text{MS2}\right] \end{array}$$ (44)

$$\begin{array}{ccc} BF(s) & = & \exp(-E(s)/RT) \end{array}$$ (45)

$$\begin{array}{ccc} Q_{i,j} & = & \sum_{s\in SS[i,j]}BF\left(s\right)\cdot N\left(s\right) \end{array}$$ (46)

$$\begin{array}{ccc} QB_{i,j} & = & \sum_{s\in SS[i,j];(i,j)\in s}BF\left(s\right)\cdot N\left(s\right) \end{array}$$ (47)

$$\begin{array}{ccc} Z_{i,j} & = & \sum_{s\in SS[i,j]}\exp\left(-E\left(s\right)/RT\right) \end{array}$$ (48)

$$\begin{array}{ccc} ZB_{i,j} & = & \sum_{s\in SS[i,j];(i,j)\in s}\exp\left(-E\left(s\right)/RT\right) \end{array}$$ (49)

Note that *I* is the indicator function, and that *QB*_*i,j*_ is the Boltzmann weighted sum of the number of neighbors, using move set MS2, where the sum is taken over all structures s∈SS[i,j] that contain the base pair (*i, j*). Similarly *ZB*_*i,j*_ is the sum of Boltzmann factors *BF*(*s*), where the sum is taken over all structures s∈SS[i,j] that contain the base pair (*i, j*). We write *bp*(*k, j*) = 1 to mean that nucleotides *a*_*k*_, *a*_*j*_ can form either a Watson-Crick or wobble base pair, and for nucleotide *c* ∈ {*A, C, G, U*}, we write *bp*(*k, c*) = 1 to mean that nucleotides *a*_*k*_ and *c* can form a Watson-Crick or wobble base pair. From the context, there should be no confusion between *bp*(*k, j*) and *bp*(*k, c*).

#### Auxilliary functions *EL, ER, ER*′, *F, G*

For 1 ≤ *i* ≤ *j* ≤ *n, c* ∈ {*A, C, G, U*}, and *x* ∈ [0, *n*], and *c* ∈ {*A, C, G, U*}, define the Boltzmann version of the functions defined in the previous Section “Uniform, non-homopolymer Model B”, where without risk of confusion we use the same function notations for *EL*_*i,j,c*_, *ER*_*i,j,c*_, $ER_{i,j,c}^{\prime }$, *F*_*i,j,c,x*_, *G*_*i,j,c,x*_, although the underlying definitions must be modified.

$$\begin{array}{ccc} EL_{i,j,c} & = & \sum_{s\in SS[i,j]}\sum_{\left(x,y\right)}BF\left(s\right)\cdot I[\left(x,y\right)\;\text{is}\;\text{an}\;\text{external}\;\text{base}\;\text{pair}\;\text{(bp)}\;\text{in}\;s\text{,}\;bp(x,c)=1] \end{array}$$ (50)

$$\begin{array}{ccc} ER_{i,j,c} & = & \sum_{s\in SS[i,j]}\sum_{\left(x,y\right)}BF\left(s\right)\cdot I[\left(x,y\right)\;\text{is}\;\text{external}\;\text{bp}\;\text{in}\;s\text{,}\;bp(y,c)=1] \end{array}$$ (51)

$$\begin{array}{ccc} ER_{i,j,c}^{\prime } & = & \sum_{\begin{array}{c} s\in SS[i,j]\\ (x,y)\in s\; \end{array}}BF\left(s\right)\cdot I[(x,y)\in s\;\text{is}\;\text{ext.}\;\text{bp}\;\text{in}\;s\text{,}\;bp(y,c)=1\text{,}\;y\leq j-\theta -1\text{,}\;j\;\text{unpaired}\;\text{in}\;s\;] \end{array}$$ (52)

$$\begin{array}{ccc} F_{i,j,c,x} & = & \sum_{s\in SS[i,j]}BF\left(s\right)\cdot I[s\;\text{has}\;x\;\text{visible}\;\text{occurrences}\;\text{of}\;\text{a}\;\text{nucleotide}\;\text{that}\;\text{can}\;\text{pair}\;\text{with}\;c] \end{array}$$ (53)

$$\begin{array}{ccc} G_{i,j,c,x} & = & \sum_{s\in SS[i,j]}BF\left(s\right)\cdot I[s\;\text{has}\;\text{exactly}\;x\;\text{visible}\;\text{occurrences}\;\text{of}\;\text{a}\;\text{nucleotide}\;\text{in}\;\left[1,j-\theta -1\right]\\ & & \text{that}\;\text{can}\;\text{pair}\;\text{with}\;c\text{,}\;\text{and}\;j\;\text{unpaired}\;\text{in}\;s] \end{array}$$ (54)

Recursions for a dynamic programming implementation of these functions are given later in Section “Recursions for auxilliary functions”. We focus now on how to compute *Q*_*i,j*_ using these auxilliary functions.

#### Recursion for function *Q*_*i,j*_

For notational convenience, define *Q*_*i,i* − 1_ = 0 and *Z*_*i,i*−1_ = 1 for all 1 ≤ *i* ≤ *n*. If *i* ≤ *j* < *i* + *θ* + 1, then for any secondary structure s∈SS[i,j], there are no structural neighbors of *s* and so *Q*_*i,j*_ = 0. If *i* ≤ *j* < *i* + *θ* + 1, then the only secondary structure on [*i, j*] is the empty structure with free energy of zero, so *Z*_*i,j*_ = 1. Now assume that *i* + *θ* + 1 ≤ *j*. By definition

$$\begin{array}{ccc} Q_{i,j} & = & \sum_{\begin{array}{c} s\in SS[i,j]\\ j\;\text{unpaired}\;\text{in}\;s \end{array}}BF\left(s\right)N\left(s\right)+\sum_{k=i}^{j-\theta -1}\sum_{\begin{array}{c} s\in SS[i,j]\\ (k,j)\in s \end{array}}BF\left(s\right)N\left(s\right). \end{array}$$ (55)

For the move set MS1 (in the absence of shift moves), it has been shown in [34] that

$$\begin{array}{ccc} Q_{i,j} & = & Q_{i,j-1}+\sum_{k=i}^{j-\theta -1}bp\left(k,j\right)\cdot (Z_{i,k-1}\cdot Z_{k+1,j-1}+Q_{i,k-1}\cdot ZB_{k,j}+Z_{i,k-1}\cdot QB_{k,j}) \end{array}$$ (56)

However, when allowing shift moves, the situation is more complicated since there are shifts involving *x, y, x*′, *y*′ ∈ [*i, j*] that are neither fully contained in the segment [*i, j* − 1] for structures s∈SS[i,j] in which *j* is unpaired, nor fully contained in one of the segments [*i, k* − 1], [*k, j*] structures s∈SS[i,j] which contain the base pair (*k, j*). The former shifts are treated in cases 1(c), 1(d), while the latter shifts are treated in cases 2(c), 2(d).

For clarity in the derivation of *Q*_*i,j*_, we start by explicitly listing the moves in move set MS2. Let *x, z*′, *y, y*′ denote distinct positions all belonging to the interval [*i, j*]. The structure *t* can be obtained from structure *s* by a move from MS2, if *t* is a valid secondary structure and can be obtained from *s* by applying a move of the form 1–6.

1. Addition of a base pair (*x, y*) to *s*.
2. Removal of a base pair (*x, y*) from *s*.
3. Shift of a base pair (*x, y*) in *s* to (*x, y*′) in *t*.
4. Shift of a base pair (*x, y*) in *s* to (*y*′, *x*) in *t*.
5. Shift of a base pair (*x, y*) in *s* to (*x*′, *y*) in *t*.
6. Shift of a base pair (*x, y*) in *s* to (*y, x*′) in *t*.

The shift moves 3–6 are depicted in Fig 8. Notice that in shifts of type 3, 4 the original position *x* is retained, while in shifts of type 5, 6 the original position *y* is retained. for distinct *x, x*′, *y* in the interval [*i, j*].

In the base case, for all *i* ∈ [1, *n*], we have *Q*_*i,i* − 1_ = 0, *Z*_*i,i* − 1_ = 1, and for *i* ≤ *j* ≤ *i* + *θ* = *i* + 3, *Q*_*i*,*j*_ = 0, *Z*_*i*,*j*_ = 1. For the inductive case in which *j* − *i* > *θ* = 3, initialize *Q*_*i*,*j*_ = 0 and then add the contributions from the cases below. The recursions for *Z*_*i*,*j*_ are well-known [39] and are given later in Section “Remaining recursions for *Q*_*i*,*j*_ and *Z*_*i*,*j*_”.

Case 1(a): In this case, we consider the contribution from s∈SS[i,j], in which *j* is unpaired in the interval [*i, j*], and *t* is obtained from *s* by a move from MS2 involving *x, y, x*′, *y*′ ∈ [*i, j* − 1]. The contribution is

$$\begin{array}{ccc} Q_{i,j} & += & Q_{i,j-1}. \end{array}$$ (57)

which accounts for the addition, removal or shift of a base pair in [*i, j* − 1]. Note that shifts of base pairs involving the last position *j* are not considered in Case 1(a)—such shifts will treated in cases 1(c), 1(d), 2(c), 2(d).

Case 1(b): In this case, we consider the contribution from s∈SS[i,j], in which *j* is unpaired in [*i, j*], and *t* is obtained from *s* by adding the base pair (*k, j*) for some *i* ≤ *k* ≤ *j* − *θ* − 1 = *j* − 4. The contribution is

$$\begin{array}{ccc} Q_{i,j} & += & \sum_{k=i}^{j-\theta -1}bp\left(k,j\right)\cdot Z_{i,k-1}\cdot Z_{k+1,j-1}. \end{array}$$ (58)

This term arises from those *t* obtained from *s* by adding a base pair (*k, j*) for some *k* ∈ [*i, j* − *θ* − 1].

The remaining cases 1(c), 1(d) treat shifts involving *x, y, x*′, *y*′ ∈ [*i, j*] in structures s∈SS[i,j] in which *j* is unpaired in [*i, j*], where the position *j* is *touched*; i.e. it is not the case that *x, y, x*′, *y*′ ∈ [*i, j* − 1] and so these shifts are not already counted in the term *Q*_*i,j* − 1_.

Case 1(c): In this case, depicted in panel (a) of Fig 9, we consider the contribution from s∈SS[i,j] in which *j* is unpaired in [*i, j*], and *t* is obtained from *s* by a shift of the base pair (*x, y*) to (*x, j*) for *i* ≤ *x* ≤ *y* − *θ* − 1 and *y* ≤ *j* − 1. The function *EL*_*i*,*j* − 1,*a*_*j*__ is the sum, taken over all structures s∈SS[i,j] in which *j* in unpaired, of the product of the Boltzmann factor *B*(*s*) times the number of external base pairs (*x, y*) in *s* with *y* ≤ *j* − 1 such that the nucleotide *a*_*x*_ at position *x* can form a base pair with the nucleotide *a*_*j*_ at position *j*. For any such (*x, y*), it is possible to shift the base pair (*x, y*) to (*x, j*), and vice versa. Before proceeding, note that the current Case 1(c) handles shifts from (*x, y*) to (*x, j*), while Case 2(b) handles shifts from (*x, j*) to (*x, y*). The contribution in the current case is clearly

$$\begin{array}{ccc} Q_{i,j} & += & EL_{i,j-1,a_{j}}. \end{array}$$ (59)

Case 1(d): In this case, depicted in panel (b) of Fig 9, we consider the contribution from s∈SS[i,j] in which *j* is unpaired in [*i, j*], and *t* is obtained from *s* by a shift of the base pair (*x, y*) to (*y, j*) for *i* ≤ *x* ≤ *y* − *θ* − 1 and *y* ≤ *j* − *θ* − 1. The function $ER_{i,j,a_{j}}^{\prime }$ is the sum, taken over all structures s∈SS[i,j] in which *j* in unpaired, of the product of the Boltzmann factor *B*(*s*) times the number of external base pairs (*x, y*) in *s* with *y* ≤ *j* − *θ* − 1 such that the nucleotide *a*_*y*_ at position *y* can form a base pair with the nucleotide *a*_*j*_ at position *j*. For any such external base pair (*x, y*), it is possible to shift (*x, y*) to (*y, j*), and vice versa. Before proceeding, note that the current Case 1(d) handles shifts from (*x, y*) to (*y, j*), while Case 2(d) handles shifts from (*y, j*) to (*x, y*). The contribution in the case at hand is clearly

$$\begin{array}{ccc} Q_{i,j} & += & ER_{i,j,a_{j}}^{\prime }. \end{array}$$ (60)

Case 2(a): In this case, we consider the contribution from structures s∈SS[i,j], which contain the base pair (*k, j*), for some *i* ≤ *k* ≤ *j* − *θ* − 1, and *t* is obtained from *s* by a move from MS2 involving *x, y, x*′, *y*′, such that *x, y, x*′, *y*′ ∈ [*i, k* − 1]. The contribution is

$$\begin{array}{ccc} Q_{i,j} & += & \sum_{k=i}^{j-\theta -1}bp\left(k,j\right)\cdot Q_{i,k-1}\cdot ZB_{k,j}. \end{array}$$ (61)

Case 2(b): In this case, we consider the contribution from structures s∈SS[i,j], which contain the base pair (*k, j*), for some *i* ≤ *k* ≤ *j* − *θ* − 1, and *t* is obtained from *s* by a move from MS2 involving *x, y, x*′, *y*′, such that *x, y, x*′, *y*′ ∈ [*k, j*]. The contribution is

$$\begin{array}{ccc} Q_{i,j} & += & \sum_{k=i}^{j-\theta -1}bp\left(k,j\right)\cdot Z_{i,k-1}\cdot QB_{k,j}. \end{array}$$ (62)

The remaining cases 2(c), 2(d) treat shifts involving *x, y, x*′, *y*′ ∈ [*i, j*] in structures s∈SS[i,j] which contain the base pair (*k, j*) for some *i* ≤ *k* ≤ *j* − *θ* − 1, where it is neither the case that *x, y, x*′, *y*′ ∈ [*i, k* − 1] nor *x, y, x*′, *y*′ ∈ [*k, j*]; i.e. cross talk shifts that *touch* both the left [*i, k* − 1] and the right [*k, j*] segments.

Case 2(c): In this case, depicted in panel (c) of Fig 9, we consider the contribution from s∈SS[i,j], which contain the base pair (*k, j*), for some *i* ≤ *k* ≤ *j* − *θ* − 1, and *t* is obtained from *s* by a shift of the base pair (*k, j*) to (*k*′, *j*) for some *k*′ < *k* that is *visible* in structure *s*\{(*k, j*)}. Before proceeding, note that for *k* < *k*′, the shift of base pair (*k, j*) to (*k*′, *j*) is treated in Case 2(b).

Recall that the function *F*_*i*,*k* − 1,*a*_*j*_, *x*_ is the sum of Boltzmann factors of all structures *s*_0_ on [*i, k* − 1] that contain exactly *x* occurrences of a visible position that can form a base pair with the nucleotide *a*_*j*_ at position *j*. The contribution in this case is

$$\begin{array}{ccc} Q_{i,j} & += & \sum_{k=i}^{j-\theta -1}\sum_{x=1}^{k-i}bp\left(k,j\right)\cdot x\cdot F_{i,k-1,a_{j},x}\cdot ZB_{k,j}. \end{array}$$ (63)

Case 2(d): In this case, depicted in panel (d) of Fig 9, we consider the contribution from structures s∈SS[i,j], which contain the base pair (*k, j*), for some *i* ≤ *k* ≤ *j* − *θ* − 1, and *t* is obtained from *s* by a shift of the base pair (*k, j*) to (*k*′, *k*) for some *i* ≤ *k*′ ≤ *k* − *θ* − 1 which is *visible* in *s*. Recall that the function *G*_*i,k,a*_*k*_, *x*_ is the sum of Boltzmann factors of all structures *s*_0_ on [*i, k*], in which *k* is unpaired, for which there are exactly *x* occurrences of a visible position in [*i, k* − *θ* − 1] that can form a base pair with *a*_*k*_. The contribution is

$$\begin{array}{ccc} Q_{i,j} & += & \sum_{k=i}^{j-\theta -1}\sum_{x=1}^{k-i}bp\left(k,j\right)\cdot x\cdot G_{i,k,a_{k},x}\cdot ZB_{k,j}. \end{array}$$ (64)

Putting together all contributions from Case 1(a) through Case 2(d), we have

$$\begin{array}{ccc} Q_{i,j} & = & Q_{i,j-1}+\sum_{k=i}^{j-\theta -1}bp\left(k,j\right)\cdot (Z_{i,k-1}\cdot Z_{k+1,j-1}+Q_{i,k-1}\cdot ZB_{k,j}+Z_{i,k-1}\cdot QB_{k,j})+\\ & & EL_{i,j-1,a_{j}}+ER_{i,j,a_{j}}^{\prime }+\sum_{k=i}^{j-\theta -1}\sum_{x=1}^{k-i}bp\left(k,j\right)\cdot x\cdot (F_{i,k-1,a_{j},x}+G_{i,k,a_{k},x})\cdot ZB_{k,j} \end{array}$$ (65)

#### Recursions for auxilliary functions

We now provide the recursions for functions *EL, ER, ER*′, *F* and *G*.

#### Definition of *EL*

For 1 ≤ *i* ≤ *j* ≤ *n* and *c* ∈ {*A, C, G, U*}, we define *EL*_*i,j,c*_ by induction on *j* − *i*, where

$$\begin{array}{ccc} EL_{i,j,c} & = & \sum_{s\in SS[i,j]}\sum_{\left(x,y\right)}BF\left(s\right)\cdot I[\left(x,y\right)\;\text{is}\;\text{external}\;\text{bp}\;\text{in}\;s\text{,}\;bp(x,c)=1] \end{array}$$ (66)

Base Case: If *j* − *i* ≤ *θ*, define *EL*_*i,j,c*_ = 0.

Inductive Case: If *j* − *i* > *θ*, define *EL*_*i,j,c*_ as the sum of the following

$$\begin{array}{ccc} EL_{i,j,c} & = & EL_{i,j-1,c}+bp\left(i,j\right)\cdot bp\left(i,c\right)\cdot ZB_{i,j}+\sum_{k=i+1}^{j}bp\left(k,j\right)\cdot EL_{i,k-1,c}\cdot ZB_{k,j}+\\ & & \sum_{k=i+1}^{j}bp\left(k,j\right)\cdot bp\left(k,c\right)\cdot Z_{i,k-1}\cdot ZB_{k,j} \end{array}$$ (67)

#### Definition of *ER*

For 1 ≤ *i* ≤ *j* ≤ *n* and *c* ∈ {*A, C, G, U*}, we define *ER*_*i,j,c*_ by induction on *j* − *i*, where

$$\begin{array}{ccc} ER_{i,j,c} & = & \sum_{s\in SS[i,j]}\sum_{\left(x,y\right)}BF\left(s\right)\cdot I[\left(x,y\right)\;\text{is}\;\text{external}\;\text{bp}\;\text{in}\;s\text{,}\;bp(y,c)=1] \end{array}$$ (68)

Base Case: If *j* − *i* ≤ *θ*, define *ER*_*i,j,c*_ = 0.

Inductive Case: If *j* − *i* > *θ*, define *ER*_*i,j,c*_ as the sum of the following

$$\begin{array}{ccc} ER_{i,j,c} & = & ER_{i,j-1,c}+bp\left(i,j\right)\cdot bp\left(j,c\right)\cdot ZB_{i,j}+\sum_{k=i+1}^{j}bp\left(k,j\right)\cdot ER_{i,k-1,c}\cdot ZB_{k,j}+\\ & & \sum_{k=i+1}^{j}bp\left(k,j\right)\cdot bp\left(j,c\right)\cdot Z_{i,k-1}\cdot ZB_{k,j} \end{array}$$ (69)

#### Definition of *ER*′

For 1 ≤ *i* ≤ *j* ≤ *n* and *c* ∈ {*A, C, G, U*}, we define $ER_{i,j,c}^{\prime }$ by induction on *j* − *i*, where

$$\begin{array}{ccc} ER_{i,j,c}^{\prime } & = & \sum_{s\in SS[i,j]}\sum_{\left(x,y\right)}BF\left(s\right)\cdot \\ & & I[(x,y)\in s\;\text{is}\;\text{external}\;\text{bp}\;\text{in}\;s\text{,}\;bp(y,c)=1\text{,}\;y\leq j-\theta -1\text{,}\;j\;\text{unpaired}\;\text{in}\;s\;] \end{array}$$ (70)

Base Case: If *j* − *i* ≤ *θ*, define $ER_{i,j,c}^{\prime }=0$.

Inductive Case: If *j* − *i* > *θ*, define $ER_{i,j,c}^{\prime }$ as the sum of the following

$$\begin{array}{ccc} ER_{i,j,c}^{\prime } & = & ER_{i,j-\theta -1,c}+\\ & & \sum_{u=1}^{\theta }\sum_{k=i+1}^{j-\theta -1+u-\theta -1}bp\left(k,j-\theta -1+u\right)\cdot I\left[j-\theta -1+u-k>\theta \right]\cdot ER_{i,k-1,c}\cdot ZB_{k,j-\theta -1+u} \end{array}$$ (71)

Note that the first term to the right of the equality sign in the previous equation is *ER*_*i*,*j* − *θ* − 1, *c*_ and *not* $ER_{i,j-\theta -1,c}^{\prime }$.

#### Definition of *F*

For 1 ≤ *i* ≤ *j* ≤ *n, c* ∈ {*A, C, G, U*} and *x* ∈ [0, *n*], we define *F*_*i,j,c, x*_ by induction on *j* − *i*, where

$$\begin{array}{ccc} F_{i,j,c,x} & = & \sum_{s\in SS[i,j]}BF\left(s\right)\cdot I[s\;\text{has}\;\text{exactly}\;x\;\text{visible}\;\text{occurrences}\;\text{of}\;\text{a}\;\text{base}\;\text{that}\;\text{can}\;\text{pair}\;\text{with}\;c] \end{array}$$ (72)

Define *F*_*i,j,c,x*_ = 0 for *j* < *i* and *c* ∈ {*A, C, G, U*} and *x* ∈ [0, *n*].

Base Case *i* = *j*: For *c* ∈ {*A, C, G, U*}, define *F*_*i,i,c,bp*(*i, c*)_ as follows

$$\begin{array}{ccc} F_{i,i,c,0} & = & \left\{ \begin{array}{cc} 1 & \text{if}\;bp(i,c)=0\\ 0 & \text{else} \end{array}\right. \end{array}$$ (73)

and

$$\begin{array}{ccc} F_{i,i,c,1} & = & \left\{ \begin{array}{cc} 1 & \text{if}\;bp(i,c)=1\\ 0 & \text{else} \end{array}\right. \end{array}$$ (74)

Base Case *i* < *j* ≤ *i* + *θ*: For *i* < *j* ≤ *i* + *θ*, and *x* ∈ [0, *j* − *i* + 1], define by double induction on *j* − *i* and *x*

$$\begin{array}{ccc} F_{i,j,c,x} & = & \left\{ \begin{array}{cc} F_{i,j-1,c,x-1} & \text{if}\;x>0\;\text{and}\;bp(j,c)=1\\ F_{i,j-1,c,x} & \text{if}\;bp(j,c)=0 \end{array}\right. \end{array}$$ (75)

Inductive Case *j* > *i* + *θ*: For *j* > *i* + *θ*, and *x* ∈ [0, *n*], we define *F* by double induction on *j* − *i* and *x*, where we separate the case that *x* = 0 and *x* > 0.

Subcase *x* = 0:

$$\begin{array}{ccc} F_{i,j,c,0} & = & \left(1-bp\left(j,c\right)\right)\cdot F_{i,j-1,c,0}+bp\left(i,j\right)\cdot ZB_{i,j}+\sum_{k=i+1}^{j-\theta -1}bp\left(k,j\right)\cdot F_{i,k-1,c,0}\cdot ZB_{k,j} \end{array}$$ (76)

Subcase *x* > 0:

$$\begin{array}{ccc} F_{i,j,c,x} & = & bp\left(j,c\right)\cdot F_{i,j-1,c,x-1}+\sum_{k=i+1}^{j-\theta -1}bp\left(k,j\right)\cdot I\left[x\in \left[0,k-i\right]\right]\cdot F_{i,k-1,c,x}\cdot ZB_{k,j} \end{array}$$ (77)

#### Definition of *G*

Recall that *G*_*i,j,c,x*_ is defined to be the sum of Boltzmann factors of structures s∈SS[i,j] having exactly *x* visible occurrences of a nucleotide in [*i, j* − *θ* − 1] that can base-pair with *c*, and *j* is unpaired in *s*, i.e.

$$\begin{array}{ccc} G_{i,j,c,x} & = & \sum_{s\in SS[i,j]}BF\left(s\right)\cdot I[s\;\text{has}\;\text{exactly}\;x\;\text{visible}\;\text{occurrences}\;\text{of}\;\text{a}\;\text{nucleotide}\;\text{in}\;\left[1,j-\theta -1\right]\\ & & \text{that}\;\text{can}\;\text{pair}\;\text{with}\;c\text{,}\;\text{and}\;j\;\text{unpaired}\;\text{in}\;s] \end{array}$$ (78)

Initially define *G*_*i,j,c,x*_ = 0 for all *i, j, c, x*.

Base Case: For *i* ≤ *j* ≤ *i* + *θ*, and *c* ∈ {*A, C, G, U*}, define *G*_*i,j,c*, 0_ = 0.

Inductive Case: In this case, *j* > *i* + *θ*, and *c* ∈ {*A, C, G, U*}. We separately treat the subcases *x* = 0 and *x* > 0.

Subcase *x* = 0:

$$\begin{array}{ccc} G_{i,j,c,0} & = & F_{i,j-\theta -1,c,0}+\sum_{u=1}^{3}I\left[j-\theta -1+u-i>\theta \right]\cdot bp\left(i,j-\theta -1+u\right)\cdot ZB_{i,j-\theta -1+u}+\\ & & \sum_{u=1}^{3}\sum_{k=i+1}^{j-\theta -1+u-\theta -1}I\left[j-\theta -1+u-k>\theta \right]\cdot bp\left(k,j-\theta -1+u\right)\cdot F_{i,k-1,c,0}\cdot ZB_{k,j-\theta -1+u} \end{array}$$ (79)

Subcase *x* > 0:

$$\begin{array}{ccc} G_{i,j,c,x} & = & F_{i,j-\theta -1,c,x}+\\ & & \sum_{u=1}^{3}\sum_{k=i+1}^{j-\theta -1+u-\theta -1}I\left[j-\theta -1+u-k>\theta \right]\cdot bp\left(k,j-\theta -1+u\right)\cdot F_{i,k-1,c,x}\cdot ZB_{k,j-\theta -1+u} \end{array}$$ (80)

#### Remaining recursions for *Q*_*i*,*j*_ and *Z*_*i*,*j*_

In this section, we furnish the remaining recursions for *Q*_*i*,*j*_, *Z*_*i*,*j*_ in the Turner 2004 energy model [36]. For a fixed sequence **a** = **a**_1_, …, **a**_*n*_ and for 1 ≤ *i* ≤ *j* ≤ *n*, define

$$\begin{array}{ccc} Q_{i,j} & = & \sum_{s\in SS[i,j]}N_{s}\cdot \exp\left(-E\left(s\right)/RT\right)\\ Z_{i,j} & = & \sum_{s\in SS[i,j]}\exp\left(-E\left(s\right)/RT\right) \end{array}$$ (81)

where *N*_*s*_ is the number of secondary structures that can be obtained from *s* by a base pair addition, removal or shift–i.e. the number of neighbors of *s* with respect to move set MS2. It follows that *Z* = *Z*_1, *n*_ is the partition function for secondary structures, and

$$\begin{array}{c} \left\langle N_{s}\right\rangle =\frac{Q_{1,n}}{Z_{1,n}}=\sum_{s\in SS[1,n]}N_{s}\cdot P\left(s\right)=\sum_{s\in SS[1,n]}N_{s}\cdot \frac{\exp(-E(s)/RT)}{Z}=\sum_{s\in SS[1,n]}N_{s}\cdot \frac{BF(s)}{Z} \end{array}$$ (82)

where *BF*(*s*) abbreviates the Boltzmann factor exp(−*E*(*s*)/*RT*) of *s*.

To provide a self-contained treatment, we recall McCaskill’s algorithm [39], which efficiently computes the partition function. For RNA nucleotide sequence **a** = **a**_1_, …, **a**_*n*_, let *H*(*i, j*) denote the free energy of a hairpin closed by base pair (*i, j*), while *IL*(*i, j, i*′, *j*′) denotes the free energy of an *internal loop* enclosed by the base pairs (*i, j*) and (*i*′, *j*′), where *i* < *i*′ < *j*′ < *j*. Internal loops comprise the cases of stacked base pairs, left/right bulges and proper internal loops. The free energy for a multiloop containing *N*_*b*_ base pairs and *N*_*u*_ unpaired bases is given by the affine approximation *a* + *bN*_*b*_ + *cN*_*u*_.

**Definition 2 (Partition function *Z* and related function *Q*)**

- *Z*_*i*,*j*_ = ∑_*s*_ exp(−*E*(*s*)/*RT*) *where the sum is taken over all structures* s∈SS[i,j].
- *ZB*_*i*,*j*_ = ∑_*s*_ exp(−*E*(*s*)/*RT*) *where the sum is taken over all structures* s∈SS[i,j] *which contain the base pair* (*i, j*).
- *ZM*_*i*,*j*_ = ∑_*s*_ exp(−*E*(*s*)/*RT*) *where the sum is taken over all structures* s∈SS[i,j] *which are contained within an enclosing multiloop having* at least *one component*.
- *ZM*1_*i*,*j*_ = ∑_*s*_ exp(−*E*(*s*)/*RT*) *where the sum is taken over all structures* s∈SS[i,j] *which are contained within an enclosing multiloop having* exactly *one component. Moreover, it is* required *that (i, r) is a base pair of x, for some i < r ≤ j*.
- *Q*_*i*,*j*_ = ∑_*s*_ *N*_*s*_ ⋅ exp(−*E*(*s*)/*RT*) *where the sum is taken over all structures* s∈SS[i,j].
- *QB*_*i*,*j*_ = ∑_*s*_ *N*_*s*_ ⋅ exp(−*E*(*s*)/*RT*) *where the sum is taken over all structures* s∈SS[i,j] *which contain the base pair (i, j)*.
- *QM*_*i*,*j*_ = ∑_*s*_ *N*_*s*_ ⋅ exp(−*E*(*s*)/*RT*) *where the sum is taken over all structures* s∈SS[i,j] *which are contained within an enclosing multiloop having* at least *one component*.
- *QM*1_*i*,*j*_ = ∑_*s*_ *N*_*s*_ ⋅ exp(−*E*(*s*)/*RT*) *where the sum is taken over all structures* s∈SS[i,j] *which are contained within an enclosing multiloop having* exactly *one component. Moreover, it is* required *that (i, r) is a base pair of s, for some i* < *r* ≤ *j*.

We will define *Z*_*i*,*j*_ and *Q*_*i*,*j*_ by recursion on *j* − *i*, for 1 ≤ *i* ≤ *j* ≤ *n*.

Base Case: Recalling that *θ* = 3, for *j* − *i* ∈ {−1, 0, 1, 2, 3}, define *Q*_*i*,*j*_ = *QB*_*i*,*j*_ = 0, *Z*_*i*,*j*_ = 1, *ZB*_*i*,*j*_ = *ZM*_*i*,*j*_ = *ZM*1_*i*,*j*_ = 0, since the empty structure is the only possible secondary structure.

Inductive Case for *Z*_*i*,*j*_: For *j* > *i* + *θ*, define

$$\begin{array}{ccc} Z_{i,j} & = & Z_{i,j-1}+ZB_{i,j}+\sum_{r=i+1}^{j-\theta -1}Z_{i,r-1}\cdot ZB_{r,j} \end{array}$$ (83)

$$\begin{array}{ccc} ZB_{i,j} & = & \exp\left(-H\left(i,j\right)/RT\right)+\sum_{i\leq \ell \leq r\leq j}\exp\left(-IL\left(i,j,\ell ,r\right)/RT\right)\cdot ZB_{\ell ,r}+\\ & & \exp\left(-\left(a+b\right)/RT\right)\cdot (\sum_{r=i+\theta +1}^{j-\theta -2}ZM_{i+1,r-1}\cdot ZM1_{r,j-1}) \end{array}$$ (84)

$$\begin{array}{ccc} ZM1_{i,j} & = & \sum_{r=i+\theta +1}^{j}ZB_{i,r}\cdot \exp\left(-c\left(j-r\right)/RT\right) \end{array}$$ (85)

$$\begin{array}{ccc} ZM_{i,j} & = & \sum_{r=i}^{j-\theta -1}ZM1_{r,j}\cdot \exp\left(-\left(b+c\left(r-i\right)\right)/RT\right)+\\ & & \sum_{r=i+\theta +2}^{j-\theta -1}ZM_{i,r-1}\cdot ZM1_{r,j}\cdot \exp\left(-b/RT\right). \end{array}$$ (86)

Inductive Case for *Q*_*i*,*j*_: For *j* > *i* + *θ*, recall that by Eq (65) we have

$$\begin{array}{ccc} Q_{i,j} & = & Q_{i,j-1}+\sum_{k=i}^{j-\theta -1}bp\left(k,j\right)\cdot (Z_{i,k-1}\cdot Z_{k+1,j-1}+Q_{i,k-1}\cdot ZB_{k,j}+Z_{i,k-1}\cdot QB_{k,j})+\\ & & EL_{i,j-1,a_{j}}+ER_{i,j,a_{j}}^{\prime }+\sum_{k=i}^{j-\theta -1}\sum_{x=1}^{k-i}bp\left(k,j\right)\cdot x\cdot (F_{i,k-1,a_{j},x}+G_{i,k,a_{k},x})\cdot ZB_{k,j} \end{array}$$ (87)

To complete the definition of *QB*_*i*,*j*_, we need additional auxilliary functions.

#### Auxilliary function *arc*

To complete the inductive definition of *Q*_*i*,*j*_ just given, we must define *QB*_*i*,*j*_, *QM*1_*i*,*j*_, *QM*_*i*,*j*_. This first requires the following auxilliary definitions, which count the number of structures obtained by adding a base pair within a hairpin, bulge, internal loop or multiloop, or by shifting a base pair at a boundary of the loop. For *θ* = 3 and *j* − *i* > *θ* define

$$\begin{array}{ccc} arc1_{a}\left(i,j\right) & = & \left|\{(x,y):bp(x,y)=1,i\leq x<y\leq j,x+\theta <y\}\right|\\ arc1_{b}\left(i,j\right) & = & \left|\{(i,k):bp(i,k)=1,i<k<j,i+\theta <k\}\right|\\ arc1_{c}\left(i,j\right) & = & \left|\{(k,j):bp(k,j)=1,i<k<j,k+\theta <j\}\right|\\ arc2_{a}\left(i,j,\ell ,r\right) & = & \left|\{(x,y):bp(x,y)=1,i<x<\ell <r<y<j\}\right|\\ arc2_{b,1}\left(i,j,\ell ,r\right) & = & \left|\{(i,y):bp(i,y)=1,i<\ell <r<y<j\}|+|\{(i,y):bp(i,y)=1,i+\theta <y<\ell \}\right|\\ arc2_{b,2}\left(i,j,\ell ,r\right) & = & \left|\{(\ell ,y):bp(\ell ,y)=1,i<\ell <r<y<j\}|+|\{(x,\ell ):bp(x,\ell )=1,i<x<\ell -\theta \}\right|\\ arc2_{b}\left(i,j,\ell ,r\right) & = & arc2_{b,1}\left(i,j,\ell ,r\right)+arc2_{b,2}\left(i,j,\ell ,r\right)\\ arc2_{c,1}\left(i,j,\ell ,r\right) & = & \left|\{(x,j):bp(x,j)=1,i<x<\ell <r<j\}|+|\{(x,j):bp(x,j)=1,r<x<j-\theta \}\right|\\ arc2_{c,2}\left(i,j,\ell ,r\right) & = & \left|\{(x,r):bp(x,r)=1,i<x<\ell <r<j\}|+|\{(r,x):bp(r,x)=1,r+\theta <x<j\}\right|\\ arc2_{c}\left(i,j,\ell ,r\right) & = & arc2_{c,1}\left(i,j,\ell ,r\right)+arc2_{c,2}\left(i,j,\ell ,r\right)\\ arc2(i,j,\ell ,r) & = & arc2_{a}\left(i,j,\ell ,r\right)+arc2_{b}\left(i,j,\ell ,r\right)++arc2_{c}\left(i,j,\ell ,r\right)\\ arc3(i,j,\ell ,r) & = & arc1_{a}\left(i+1,\ell -1\right)+arc1_{a}\left(r+1,j-1\right)+arc2\left(i,j,\ell ,r\right)\\ arc4(i,j,k) & = & \left|\{(i,x):bp(i,x)=1,i<j<x\leq k,i+\theta <x\}\right|\\ arc5(i,j,k) & = & |\{(j,x):bp(j,x)=1,i<j<x\leq k,j+\theta <x\}|. \end{array}$$ (88)

Note that *arc*1_*a*_(*i, j*) counts the number of neighbors obtained from structure *s* by adding a base pair (*x, y*) in the interval [*i, j*]. In contrast, *arc*1_*b*_(*i, j*) [resp. *arc*1_*c*_(*i, j*)] counts the number of neighbors obtained from structure *s* by shifting the base pair (*i, j*) to (*i, k*) [resp. (*k, j*)] where *i* < *k* < *j*. The function *arc*2_*a*_(*i, j*, ℓ, *r*) counts the number of neighbors obtained from structure *s* by adding a base pair (*x, y*) in the internal loop bounded by the base pairs (*i, j*) and (ℓ, *r*) where *i* < *x* < ℓ < *r* < *y* < *j*–note that *i* + 1, …, ℓ − 1 and *r* + 1, …, *j* − 1 are unpaired in the internal loop bounded by (*i, j*) and (ℓ, *r*). In contrast, *arc*2_*b*,1_(*i, j*, ℓ, *r*) [resp. *arc*2_*b*,2_(*i, j*, ℓ, *r*)] counts the number of neighbors obtained from structure *s* by shifting the base pair (*i, j*) to (*i, y*) [resp. (ℓ, *r*) to either (*y*, ℓ) or (ℓ, *y*)] where *y* occurs in the internal loop closed on both sides by (*i, j*) and (ℓ, *r*). Similarly, *arc*2_*c*,1_(*i, j*, ℓ, *r*) [resp. *arc*2_*c*,2_(*i, j*, ℓ, *r*)] counts the number of neighbors obtained from structure *s* by shifting the base pair (*i, j*) to (*x, j*) [resp. (ℓ, *r*) to either (*r, x*) or (*x, r*)] where *x* occurs in the internal loop closed on both sides by (*i, j*) and (ℓ, *r*). Finally, *arc*2_*b*_(*i, j*, ℓ, *r*) [resp. *arc*2_*c*_(*i, j*, ℓ, *r*)] is equal to *arc*2_*b*,1_(*i, j*, ℓ, *r*) + *arc*2_*b*,2_(*i, j*, ℓ, *r*) [resp. *arc*2_*c*,1_(*i, j*, ℓ, *r*) + *arc*2_*c*,2_(*i, j*, ℓ, *r*)], and *arc*2(*i, j*, ℓ, *r*) is the sum of *arc*2_*a*_(*i, j*, ℓ, *r*), *arc*2_*b*_(*i, j*, ℓ, *r*), and *arc*2_*c*_(*i, j*, ℓ, *r*). Then *arc*3(*i, j*, ℓ, *r*) counts the number of neighbors obtained from structure *s* by either adding a base pair within the internal loop defined by (*i, j*) and (ℓ, *r*), or by shifting either (*i, j*) or (ℓ, *r*). For *i* < *j* < *k*, the function *arc*4(*i, j, k*) counts the number of neighbors obtained from structure *s* by shifting the base pair (*i, j*) to (*i, y*) for some *j* < *y* ≤ *k*, while *arc*5(*i, j, k*) counts the number of neighbors obtained from structure *s* by shifting the base pair (*i, j*) to (*j, y*) for some *j* < *y* ≤ *k*.

#### Recursion for *QB*_*i*,*j*_

We can now proceed with the definition of *QB*_*i*,*j*_, defined to be the sum of *A*_*i*,*j*_, *B*_*i*,*j*_, *C*_*i*,*j*_, each of which is defined below.

Case A: (*i, j*) closes a hairpin.

In this case, the contribution to *QB*_*i*,*j*_ is given by

$$\begin{array}{ccc} A_{i,j} & = & \exp(-\frac{H(i,j)}{RT})\cdot [1+arc1_{a}\left(i+1,j-1\right)+arc1_{b}\left(i,j\right)+arc1_{c}\left(i,j\right)]. \end{array}$$ (89)

The term 1 arises from the neighbor of *s* = {(*i, j*)} by removing base pair (*i, j*). The term *arc*1_*a*_(*i* + 1, *j* − 1) arises from neighbors of *s* obtained by adding a base pair in the region [*i* + 1, *j* − 1], and the term *arc*1_*b*_(*i, j*) arises from a shift of the form (*i, j*) → (*i, y*), and finally the term *arc*1_*c*_(*i, j*) arises from a shift of the form (*i, j*) → (*x, j*).

Case B: (*i, j*) closes a stacked base pair, bulge or internal loop, whose other closing base pair is (ℓ, *r*), where *i* < ℓ < *r* < *j*.

Following the convention in Vienna RNA Package, we assume that all loops have at most 30 unpaired nucleotides. This convention explains the presence of 31 in some indices. In this case, the contribution to *QB*_*i*,*j*_ is given by the following

$$\begin{array}{ccc} B_{i,j} & = & \sum_{\ell =i+1}^{\min(i+31,j-5)}\sum_{r=j-1}^{\max(j-31,i+5)}\exp(-\frac{IL(i,j,\ell ,r)}{RT})\cdot \sum_{\begin{array}{c} s\in SS[\ell ,r]\\ (\ell ,r)\in s \end{array}}BF\left(s\right)[1+arc3(i,j,\ell ,r)+N(s)]\\ & = & \sum_{\ell =i+1}^{\min(i+31,j-5)}\sum_{r=j-1}^{\max(j-31,i+5)}\exp(-\frac{IL(i,j,\ell ,r)}{RT})\cdot [ZB_{\ell ,r}\cdot (1+arc3(i,j,\ell ,r))+QB_{\ell ,r}]. \end{array}$$ (90)

The term 1 arises from the neighbor of *s* = {(*i, j*)} by removing base pair (*i, j*) (the neighbor obtained by removing base pair (ℓ, *r*) is counted by the term *N*(*s*) for s∈SS[ℓ,r]). The term *arc*3(*i, j*, ℓ, *r*) counts neighbors obtained by either adding a base pair within the internal loop defined by (*i, j*) and (ℓ, *r*), or by shifting either (*i, j*) or (ℓ, *r*).

In Case C below, we follow the convention that in the summation notation $\sum_{i=a}^{b}$, if upper bound *b* is smaller than lower bound *a*, then we intend a loop of the form: FOR *i* = *b* downto *a*.

Case C: (*i, j*) closes a multiloop.

In this case, the contribution to *QB*_*i*,*j*_ is given by the following

$$\begin{array}{ccc} C_{i,j} & = & \sum_{\begin{array}{c} s\in SS[i,j],(i,j)\in s\\ \left(i,j\right)\;\text{closes}\;\text{a}\;\text{multiloop} \end{array}}BF\left(s\right)N\left(s\right)\\ & = & \exp(-\frac{a+b}{RT})\cdot \sum_{r=i+5}^{j-5}[ZM_{i+1,r-1}\cdot ZM1_{r,j-1}+\\ & & QM_{i+1,r-1}\cdot ZM1_{r,j-1}+ZM_{i+1,r-1}\cdot QM1_{r,j-1}]. \end{array}$$ (91)

Now *QB*_*i*,*j*_ = *A*_*i*,*j*_ + *B*_*i*,*j*_ + *C*_*i*,*j*_. It nevertheless remains to define the recursions for *QM*1_*i*,*j*_ and *QM*_*i*,*j*_. These satisfy the following.

$$\begin{array}{ccc} QM1_{i,j} & = & \sum_{k=i+\theta +1}^{j}\sum_{\begin{array}{c} s\in SS[i,k]\\ (i,k)\in s \end{array}}\exp(-\frac{c(j-k)}{RT})\cdot BF\left(s\right)\cdot [N\left(s\right)+arc1_{a}\left(k+1,j\right)+arc4\left(i,k,j\right)+arc5\left(i,k,j\right)]\\ & = & \sum_{k=i+\theta +1}^{j}\exp(-\frac{c(j-k)}{RT})\cdot [QB_{i,k}+ZB_{i,k}\cdot (arc1_{a}\left(k+1,j\right)+arc4\left(i,k,j\right)+arc5\left(i,k,j\right))]. \end{array}$$ (92)

The term *arc*1_*a*_(*k* + 1, *j*) counts neighbors obtained by adding a base pair in [*k* + 1, *j*]; the term *arc*4(*i, k, j*) counts neighbors obtained by a shift of the base pair (*i, k*) to (*i, y*) for some *k* < *y* ≤ *j*; the term *arc*5(*i, k, j*) counts neighbors obtained by a shift of the base pair (*i, k*) to (*k, y*) for some *k* + *θ* < *y* ≤ *j*. Finally

$$\begin{array}{ccc} QM_{i,j} & = & \sum_{r=i}^{j-5}\exp(-\frac{b+c(r-i)}{RT})\cdot [QM1_{r,j}+ZM1_{r,j}\cdot (arc1_{a}\left(i,r-1\right)+arc1_{c}\left(i-1,r\right))]+\\ & & \sum_{r=i}^{j-5}\exp(-\frac{b}{RT})\cdot [QM_{i,r-1}ZM1_{r,j}+ZM_{i,r-1}QM1_{r,j}]. \end{array}$$ (93)

Note that in the first line of the equation for *QM*_*i*,*j*_, the position *r* is required by definition of *QM*1_*r, j*_ to pair to some position in [*r* + *θ* + 1, *j*]. Thus *r* is the left endpoint of a base pair, whose right endpoint will not be known until a subsequent call of function *QM*1_*r, j*_. The term *arc*1_*a*_(*i, r* − 1) counts neighbors obtained by adding a base pair (*x, y*) in the interval [*i, r* − 1]; the term *arc*1_*c*_(*i* − 1, *r*) counts neighbors obtained by shifting the base pair whose left endpoint is *r* to the base pair (*x, r*) for some *i* ≤ *x* < *r*. This completes the description of how to compute the expected number of neighbors with respect to the Turner energy model.

Finally, to accelerate the computation of the functions *arc*1_*a*_, …, *arc*5, the 4 × *n* × *n* array *ARC* is precomputed, where if **a** = *a*_1_, …, *a*_*n*_ denotes the input RNA sequence, then

$$\begin{array}{c} ARC\left[\alpha ,i,j\right]=\{\begin{array}{cc} |x\in \left[i,j\right]:a_{x}=U| & \text{if}\;\alpha =0\\ |x\in \left[i,j\right]:a_{x}=G| & \text{if}\;\alpha =1\\ |x\in \left[i,j\right]:a_{x}\in \left\{C,U\right\}| & \text{if}\;\alpha =2\\ |x\in \left[i,j\right]:a_{x}\in \left\{A,G\right\}| & \text{if}\;\alpha =3. \end{array} \end{array}$$ (94)

As mentioned, we follow the convention that bulges and interior loops have a size of at most 30 nt; however, this bound does not apply to hairpin loops or multiloops.

Remark: Suppose that *s* = {(*i, j*), (*i*_1_, *j*_1_), …, (*i*_*k*_, *j*_*k*_)} is a multiloop closed by (*i, j*), where *i* < *i*_1_ < *j*_1_ < *i*_2_ < *j*_2_ < ⋯ < *i*_*k*_ < *j*_*k*_ < *j*. Then note that we do not count neighbors of *s* obtained by adding a base pair (*x, y*) to the multiloop *s*, where *i* < *x* < *i*_ℓ_ < *j*_ℓ_ < *y*, nor do we count shifts within a multiloop of the form (*i*_ℓ_, *j*_ℓ_) → (*i*_ℓ_, *k*) for *j*_ℓ_ < *k*, nor (*i*_ℓ_, *j*_ℓ_) → (*k, j*_ℓ_) for *k* < *i*_ℓ_. Following the paradigm in the treatment of multiloops in McCaskill’s partition function algorithm [39], such added base pairs and shifts cannot be included. In particular, our Turner energy algorithm properly counts shifts depicted in Figs 2 and 3, but not those depicted in Fig 4. Multiloops are energetically costly due to entropic considerations, and so penalized in the Turner energy model. For this reason, multiloops are generally small, have few components, and contain few unpaired bases that might allow the formation of base pairs or support shift moves. If a multiloop has sufficient size to permit such moves, then its free energy will be large, hence the Boltzmann factor of such structures *s* is small and the contribution to ⟨*N*⟩ is negligeable. By introducing multiloop analogues of functions *EL, ER, ER*′, *F*, and *G*, it should be possible to account for such additional internal multiloop moves. However, this would lead to substantial complications of the algorithm with no likely benefit, hence this will not be pursued.

## Results

**Fig 10:**
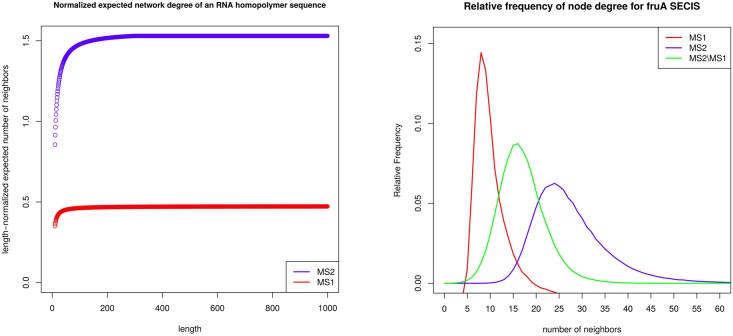
*(Left)* Normalized expected network degree of an RNA homopolymer sequence of length *n* is defined to be $\frac{Q_{n}}{nZ_{n}}$; i.e. the length-normalized expected network degree $\frac{Q_{n}}{Z_{n}}$ divided by sequence length *n*. Here *Q*_*n*_ is ∑_*s*_ *N*(*s*), where *N*(*s*) is the number of neighbors of *s*, and the sum is taken over all secondary structures *s* of the homopolymer. In the homopolymer case, the energy is 0, hence the partition function *Z*_*n*_ is simply the number of structures of the length *n* homopolymer. The purple graph was obtained with move set MS1 (base pair additions and removals), while the red graph was obtained with move set MS2 (base pair additions, removals and shifts). For *n* = 998, the value of $\frac{Q_{n}}{nZ_{n}}$ with respect to MS1 is 0.472393; using methods from enumerative combinatorics, we have analytically proved that the value of $\frac{Q_{n}}{nZ_{n}}$ with respect to MS1 is exactly 0.4734176431521986 [40]. For *n* = 998, the value of $\frac{Q_{n}}{nZ_{n}}$ with respect to MS2 is 1.530161; since the values of $\frac{Q_{n}}{nZ_{n}}$ are unchanged for *n* ≪ 998, it is likely that the asymptotic value is close to that value. It follows that there are more than 3 times more structural neighbors, on average, for move set MS2 than for move set MS1. *(Right)* Relative frequency for number of neighbors (degree) for the network of all secondary structures of the 32 nt fruA selenocysteine (SECIS) element, produced by exhaustive enumeration of all structures. The blue [resp. purple resp. red] curve corresponds to move set MS2 [resp. (MS2\MS1) resp. MS1].

In this section, we describe several results obtained by applying our novel algorithms to compute the expected network degree for given RNA sequence. The left panel of Fig 10 depicts the length-normalized expected network degree of an RNA homopolymer sequence of length *n*, defined to be $\frac{Q_{n}}{nZ_{n}}$. In the homopolymer model, *Q*_*n*_ = ∑_*s*_ *N*(*s*), where *N*(*s*) is the number of neighbors of *s*, and the sum is taken over all secondary structures *s* of [1, *n*]. In the homopolymer case, the energy is 0, so the partition function *Z*_*n*_ equals the number of structures. Fig 10 displays the normalized network degree as a function of homopolymer size, both in the case of move set MS1 (base pair additions, removals), and move set MS2 (base pair additions, removals, shifts). An asymptotic value of 0.4742 for $\frac{Q_{n}}{nZ_{n}}$ is suggested by running the dynamic programming (DP) algorithm described in Section “Homopolymer Model A” for values of sequence length 400 ≤ *n* ≤ 1000. Using methods from algebraic combinatorics, we have analytically proved that the value of $\frac{Q_{n}}{nZ_{n}}$ for MS1 is ≈ 0.4734176431521986 (see [40]). Runs of the DP algorithm also suggest that the asymptotic value of $\frac{Q_{n}}{nZ_{n}}$ for MS2 appears to be ≈ 1.530161, so that there are more than 3 times more structural neighbors, on average, for move set MS2 than for move set MS1 for the homopolymer model. The right panel of Fig 10 depicts an overlay of the degree distribution for secondary structures of the 32 nt selenocysteine element of fruA, which latter encoding the A subunit of coenzyme F420-reducing hydrogenase, for move sets MS1, MS2\MS1 and MS2.

**Fig 11:**
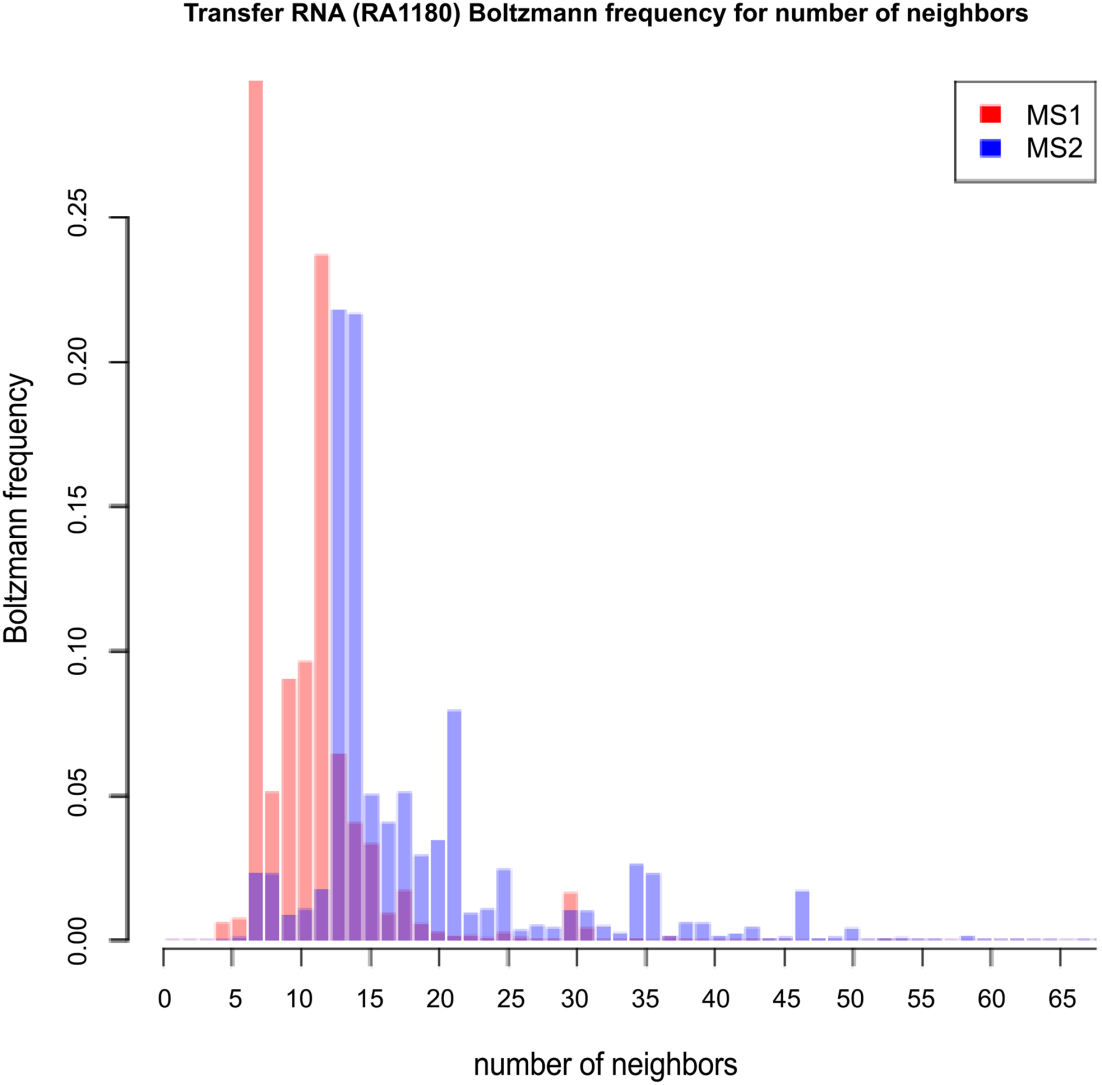
Relative frequency for the Boltzmann weighted number of neighbors for the 76 nt alanine transfer RNA from *Mycoplasma mycoides* with accession code RA1180 from tRNAdb 2009 [41], where the *sample mean* ± one standard deviation is 29.11 ± 4.63 [resp. 46.51 ± 8.74] for move set MS1 [resp. MS2] using energy model C (Turner 2004 energy parameters). The length-normalized sample mean is 0.3831 ± 0.0610 for MS1 [resp. 0.6120 ± 0.1150 for MS2]. The number of neighbors, or degree, is given on the *x*-axis. RNAsubopt -d0 -e 12 [10] was used to generate 537,180 structures *s* having free energy within 12 kcal/mol of the MFE. The sum Z* of all Boltzmann factors exp(−*E*(*s*)/*RT*) of the sampled structures was computed, and the ratio Z*/*Z* of Z* with respect to the partition function *Z* was determined to be 0.9998202. For given number *x* of neighbors, the corresponding value *y* is defined to be the sum, taken over all the structures *s*, whose degree is *x*, of the Boltzmann factor exp(−*E*(*s*)/*RT*) of *s* normalized by Z*. Using our code, with respect to energy model C (Turner 2004 energy parameters), we have the following values for the expected number of neighbors expected number of neighbors: $\frac{Q_{1,n}}{Z_{1,n}}=26.01$ (Boltzmann-MS1); $\frac{Q_{1,n}}{Z_{1,n}}=37.61$ (Boltzmann-MS2).

**Fig 12:**
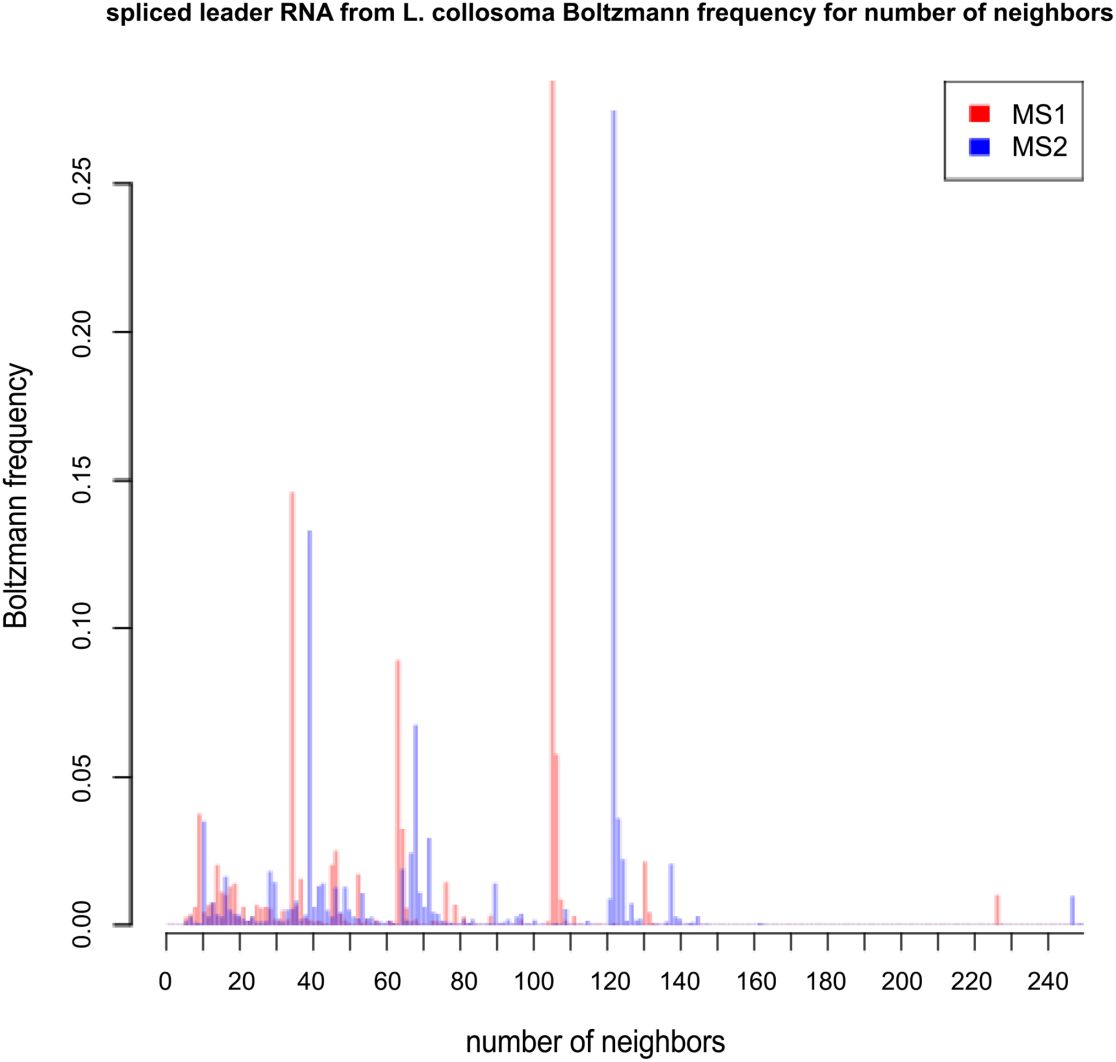
Boltzmann relative frequency for the number of neighbors for the 56 nt spliced leader RNA from *L. collosoma*, where the mean ± one standard deviation is 69.87 ± 34.04 [resp. 90.46 ± 37.71] for move set MS1 [resp. MS2] using energy model C (Turner 2004 energy parameters). The length-normalized sample mean is 1.2477 ± 0.6079 for MS1 [resp. 1.6153 ± 0.6734 for MS2]. The number of neighbors, or degree, is given on the *x*-axis. RNAsubopt -d0 -e 12 [10] was used to generate 266,065 structures *s* having free energy within 12 kcal/mol of the MFE. The sum Z* of all Boltzmann factors exp(−*E*(*s*)/*RT*) of the sampled structures was computed, and the ratio Z*/*Z* of Z* with respect to the partition function *Z* was determined to be 0.9998812, hence values of relative frequency should be close to the corresponding values for the Boltzmann probability. For given number *x* of neighbors, the corresponding value *y* is defined to be the sum, taken over all the structures *s*, whose degree is *x*, of the Boltzmann factor exp(−*E*(*s*)/*RT*) of *s* normalized by Z*. Using our code, with respect to energy model C (Turner 2004 energy parameters), we have the following values for the expected number of neighbors: $\frac{Q_{1,n}}{Z_{1,n}}=70.03$ (Boltzmann-MS1); $\frac{Q_{1,n}}{Z_{1,n}}=92.96$ (Boltzmann-MS2).

Figs 11 and 12 display the relative frequency (for energy model C) for the number of neighbors, or degree, respectively for the 76 nt alanine transfer RNA from *Mycoplasma mycoides* with accession code RA1180 from tRNAdb 2009 [41] and for the 56 nt spliced leader RNA from *L. collosoma*. RNAsubopt -d0 -e 12 [10] was used to generate 537,180 [resp. 266,065] structures *s* having free energy within 12 kcal/mol of the minimum free energy (MFE) for tRNA RA1180 [resp. spliced leader RNA from *L. collosoma*]. The sum Z* of all Boltzmann factors exp(−*E*(*s*)/*RT*) of the sampled structures was computed, and the ratio Z*/*Z* of Z* with respect to the partition function *Z* was determined to be 0.9998 for tRNA RA1180 [resp. 0.9999 for spliced leader *L. collosoma*]. For tRNA RA1180, the *sample mean* ± one standard deviation is 29.11 ± 4.63 [resp. 46.51 ± 8.74] for move set MS1 [resp. MS2] using energy model C (Turner 2004 energy parameters), while the corresponding values for *L. collosoma* spliced leader are 69.87 ± 34.04 [resp. 90.46 ± 37.71] for move set MS1 [resp. MS2]. Table 1 compares these values with those obtained by our dynamic programming method, and additionally compares values for both Turner 1999 and Turner 2004 energy parameters. Note the stark differences between the length-normalized degree distribution for transfer RNA (accession code RA1180 from tRNAdb 2009 [41]) and for the conformational switch of spliced leader from *L. collosoma*. We are currently investigating whether other conformational switches have large values of length-normalized expected number of neighbors.

**Fig 13:**
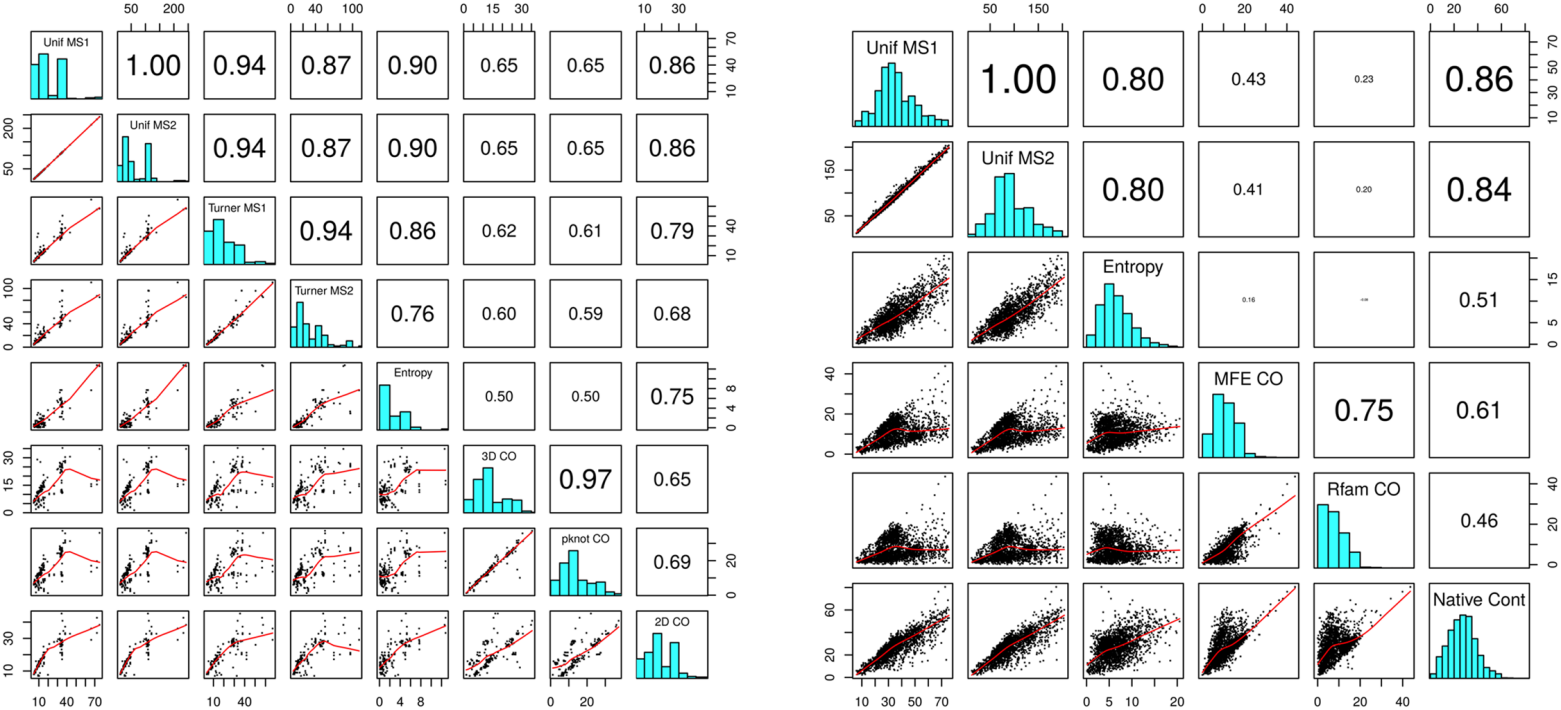
Correlation of network degree (expected number of neighbors) with (absolute) contact order, conformational entropy, expected number of native contacts, etc. determined with respect to a collection of 180 PDB files (left panel, see text) and to the first sequence with its consensus structure from the seed alignment of every family from the Rfam 12.0 database [42] (sequence length was capped at 200 nt, providing 1904 sequences and consensus structures). Move set MS1 consists of base pair additions and removals; move set MS2 consists of base pair additions, removals, and shifts. *(Left)* The rows [resp. columns] correspond to the following measures, proceeding from top to bottom [resp. left to right]: *Unif MS1*: uniform expected number of neighbors for move set MS1. *Unif MS2*: uniform expected number of neighbors for move set MS2. *Turner MS1*: Boltzmann expected number of neighbors for move set MS1. *Turner MS2*: Boltzmann expected number of neighbors for move set MS2. *Entropy*: conformational entropy −*k*_*B*_∑_*s*_ *p*(*s*) ⋅ ln*p*(*s*), where the sum is taken over all structures of a given RNA sequence, and Boltzmann probability *p*(*s*) = exp(−*E*(*s*)/*RT*)/*Z* [50]. *3D CO*: 3D (absolute) contact order, where two nucleotides are in contact if at least one atom of each is within with 6 Å. *pknot CO*: pseudoknot (absolute) contact order determined by of output of RNAview, *2D CO*: 2D CO (absolute) contact order, determined by extraction of maximal secondary structure from RNAview output. *(Right)* The rows [resp. columns] correspond to the following measures, proceeding from top to bottom [resp. left to right]: *Unif MS1, Unif MS2*, and *Entropy*: as explained in caption to left panel. *MFE CO* [resp. *Rfam CO*]: ∑_(*i, j*) ∈ *s*_0__(*j* − *i*)/∣*s*_0_∣, where the sum is taken over all base pairs (*i, j*) belonging to structure *s*_0_, and ∣*s*_0_∣ denotes the number of base pairs in *s*_0_, where *s*_0_ denotes the minimum free energy [resp. Rfam consensus] structure. *Native Cont* is number of native contacts, defined by ∑_*s*_ *P*(*s*) ⋅ ∣*s* ∩ *s*_0_∣, where the sum is taken over all structures *s, P*(*s*) = exp(−*E*(*s*)/*RT*)/*Z* is the Boltzmann probability of *s*, and ∣*s* ∩ *s*_0_∣ denotes the number of base pairs common to both *s* and *s*_0_, where *s*_0_ is the Rfam consensus structure.

Fig 13 depicts the correlation between expected network degree, conformational entropy, contact order, and expected number of native contacts, computed with respect to a collection of 180 PDB files and to a collection of 1904 RNA sequence and consensus structures taken from the Rfam 12.0 database [42]. Although the results are mixed and preliminary, the PDB data suggests a possible correlation between secondary structure *contact order* and (uniform) expected network degree, while the Rfam data suggests a possible correlation between the expected *number of native contacts* and (uniform) expected network degree. Definitions and details of the computational experiments now follow.

Contact order is considered in the context of protein folding in [43], where *absolute contact order* is defined by ∑_*i* < *j*_(*j* − *i*)/*N*, where the sum is over all *N* pairs of residues *i, j* that are in *contact*, taken here to mean that residues *i, j* each contain a heavy atom (non-hydrogen) within 6 Å, and that *i, j* are not consecutive (*j* ≠ *i* + 1). In Fig 13, we consider several formulations of RNA contact order. The *3D absolute contact order* for an RNA structure is defined as above. The *pseudoknot (pknot) absolute contact order* is defined as ∑_*i* < *j*_(*j* − *i*)/*N*, where the sum is over all *N* base pairs (*i, j*) determined by RNAview [44], a program that determines hydrogen-bonded atoms of distinct nucleotides in a PDB file of RNA and additionally classifies the base pair with respect to the Leontis-Westhof classification [45]. The *2D absolute contact order* is defined as ∑_*i* < *j*_(*j* − *i*)/*N*, where the sum is over all *N* base pairs (*i, j*) in the secondary structure extracted from RNAview output by our implementation of the method described in [46, 47], which essentially applies the Nussinov-Jacobson algorithm [48] to those base pairs determined by RNAview from the tertiary PDB structure, resulting in the secondary structure having a largest number of base pairs (one could alternatively use the web server RNApdbee [49]). We also consider the corresponding versions of *relative* contact order, by dividing the absolute contact order by RNA sequence length.

For benchmarking purposes, we took two datasets: (1) tertiary structures from the PDB, and (2) consensus secondary structures from the Rfam 12.0 database [42]. For the former, we used PDB files from the dataset [50], since these files have no discrepancies between the SEQRES and ATOM fields. From this set of 486 PDB files, we retained 180 PDB files with a total of 227 RNA chains, after removing PDB files of very short RNAs, as well as those PDB files consisting of NMR data for which RNAview [44] did not use the first MODEL in its determination of base pairing, as well as those for which RNAview returned no base pairing information at all. For the latter, we took the first sequence, with its consensus structure, from the seed alignment of every family of Rfam 12.0, where sequence length was capped at 200 nt. This provided a collection of 1904 sequences and consensus structures.

The left panel of Fig 13 depicts the correlation computed for the 180 PDB files between various formulations of *expected network degree* and RNA secondary structure *conformational entropy* [51] (highest correlation value of 0.90) and *contact order* (highest correlation value of 0.86). Here, the conformational entropy is defined by −*k*_*B*_ ⋅ ∑_*s*_ *p*(*s*) ⋅ ln*p*(*s*), where *p*(*s*) is the Boltzmann probability of secondary structure *s*, and the sum is taken over all secondary structures of a given RNA sequence (low entropy means that the Boltzmann probability is very high for a small number of structures – i.e. a relatively small number of structures has low free energy). The right panel of Fig 13 depicts the correlation for the 1904 Rfam consensus secondary structures between (uniform) *expected network degree* and various formulations of *conformational entropy* (highest correlation 0.80), the *expected number of native contacts* (highest correlation of 0.86), and two formulations of *contact order* (highest correlation value of 0.43). Here, the *expected number of native contacts* is defined by ∑_*s*_ *p*(*s*) ⋅ ∣*s* ∩ *s*_0_∣, where the sum is taken over all structures *s, p*(*s*) = exp(−*E*(*s*)/*RT*)/*Z* is the Boltzmann probability of *s*, and ∣*s* ∩ *s*_0_∣ denotes the number of base pairs common to both *s* and the Rfam consensus structure *s*_0_. At present, it is unclear why the correlation between expected network degree and contact order is higher in the PDB data than in the Rfam data.

## Conclusion

Computational methods for RNA secondary structure folding kinetics generally involve either (1) algorithms to determine optimal or near-optimal folding pathways, [6, 7, 11–13], (2) explicit solutions of the master equation for possibly coarse-grained models [14–18], or (3) repeated simulations to fold an initially empty secondary structure to the target minimum free energy (MFE) structure [5, 20–24]. Despite its importance, RNA secondary structure folding kinetics remains a computationally difficult problem, since it is known that the problem of determining optimal folding pathways is NP-complete [25].

To shed light on RNA kinetics from a different perspective, in this paper we have investigated a *network* property of RNA secondary structures. Let *G* be the network corresponding to the move set MS1 [resp. MS2] of the kinetics program Kinfold [5]; i.e. *G* = (*V, E*) is a directed graph, whose vertices are the secondary structures of a given RNA sequence and whose edges *s* → *t* are defined if structure *t* can be obtained from *s* by the addition or removal [resp. addition, removal or shift] of a base pair from *s*. In [34], we described an algorithm that computes the MS1 expected network degree ⟨*N*⟩ = ∑_*s*_ *p*(*s*) ⋅ *N*(*s*), where *N*(*s*) is the out-degree of secondary structure *s* of a user-specified RNA sequence **a** = *a*_1_, …, *a*_*n*_ and *p*(*s*) = exp(−*E*(*s*)/*RT*)/*Z* is the probability of structure *s*. In the current paper, we describe (surprisingly) much more difficult algorithms to efficiently compute the MS2 expected network degree ⟨*N*⟩ = ∑_*s*_ *p*(*s*) ⋅ *N*(*s*), with respect to increasingly complex energy models A, B, C. Model A is the *homopolymer* model [35], which we use to present a simplified version of the more complex algorithms for models B and C. Unlike the simple homopolymer model, Model B concerns the usual notion of RNA secondary structure *s*, defined in Definition 1 where the energy *E*(*s*) is zero, so that the probability *p*(*s*) is one over the number of structures (uniform probability). Model C concerns the Turner energy model without dangles, so that the probability *p*(*s*) is the Boltzmann probability of *s*; however, due to technical issues, certain low probability MS2 moves in multiloops can not be considered (see an example in Fig 4). The run time [resp. space] for our algorithm for Model A is *O*(*n* ^3^) [resp. *O*(*n* ^2^)], while that for models B and C is *O*(*n* ^4^) [resp. *O*(*n* ^3^)]—cubic space is required uniquely for functions *F, G*.

Our algorithms for Models A and B are exact, computing the same values as obtained by exhaustive brute force. Our algorithm for Model C ignores certain kinds of base pair additions, removals and shifts within a multiloop. Table 1 compares the values of expected number of neighbors (expected degree) for move sets MS1 and MS2 for Models B, C where Turner 1999 and Turner 2004 energy parameters are considered [36]. Table 1 also includes values obtained by brute force computation from structures generated by RNAsubopt [52] from the Vienna RNA Package [10]. The time required for this method is *O*(*n* ^2^) times the number of structures sampled by RNAsubopt plus the overhead to run RNAsubopt. Except for small sequences, this computation cost is prohibitive, which makes our dynamic programming computation of the expected number of neighbors an attractive alternative. Nevertheless much less information is conveyed by a single number, as shown in Table 1 than in the (approximate) distribution as shown in Fig 11 for alanine transfer RNA from *Mycoplasma mycoides* and Fig 12 for the spliced leader conformational switch from *L. collosoma*. The striking difference between these figures suggests that perhaps conformational switches may display a bimodal or multimodal degree distribution—something we are currently investigating.

**Fig 14:**
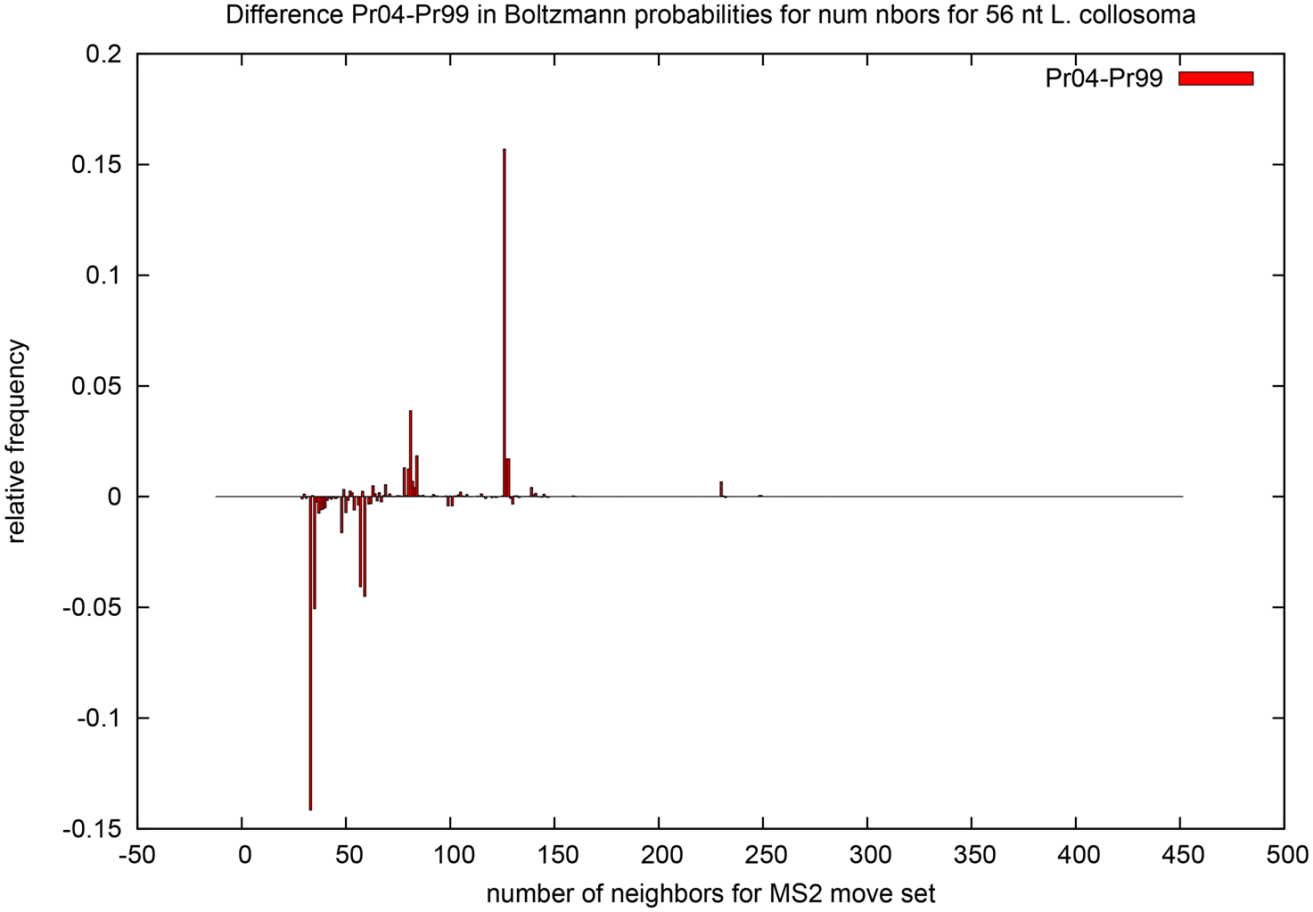
Difference in Boltzmann probabilities for 56 nt spliced leader RNA from *L. collosoma* with respect to move set MS2—see text for explanation.

Table 1 displays a strong discrepancy for the expected number of neighbors for *L. collosoma* when using Turner 1999 or Turner 2004 energy parameters. To investigate the origin of this odd discrepancy, we ran RNAsubopt -d0 -e 12 with Turner 2004 [resp. Turner 1999] parameters to generate 266,065 [resp. 259, 626] structures for 56 nt *L. collosoma* spliced leader RNA, 189, 404 of which were common to both collections. Letting Z*(04) [resp. Z*(99)] denote the sum of Boltzmann factors of these 189, 404 structures with respect to Turner 2004 [resp. Turner 1999] parameters, we computed the (pseudo) Boltzmann probability *Pr*04(*s*) = exp(−*E*04(*s*)/*RT*)/Z*(04) [resp. *Pr*99(*s*) = exp(−*E*04(*s*)/*RT*)/Z*(99)] for each of the 189, 404 common structures *s*. The difference in expected MS2 degree for Turner04 parameters minus that for Turner99 parameters is ∑_*s*_(*Pr*04(*s*)−*Pr*99(*s*)) ⋅ *N*(*s*) = 24.35. The contribution to expected degree for the set of sampled structures not common to both sets is negligeable, i.e. less than 0.01. The strongest difference between Turner04 and Turner99 values are for the 1799 [resp. 246] structures having degree 33 [resp. 126], where the difference *Pr*04(33)−*Pr*99(33) is −0.1415 [resp. 0.1570], as shown in the large negative [resp. positive] spike in Fig 14. For unknown reasons, there are striking differences in the free energy values for Turner04 and Turner99 energy models for these structures. Although the choice of Turner energy model may entail a large difference in the expected degree computed, as shown in Table 1 and Fig 14, the general form of the corresponding histograms is maintained, as shown in Figs 11 and 12. We now summarize our findings.

Given the 3D native structure of a protein, the *(absolute) contact order* is defined by ∑_*i* < *j*_(*j* − *i*)/*N*, where the sum is over all *N* pairs of residues *i, j* that are in contact, where non-contiguous residues *i, j* are in contact if each contain a heavy atom (non-hydrogen) within 6 Å [43]. We use the definition of [43] for 3D RNA contact order, whereas we define *pseudoknot (pknot) contact order* by ∑_*i* < *j*_(*j* − *i*)/*N*, where the sum is over all *N* base pairs (*i, j*) determined by RNAview [44], a program that determines hydrogen-bonded atoms of distinct nucleotides in a PDB file of RNA and additionally classifies the base pair with respect to the Leontis-Westhof classification [45]. We define *2D contact order* by ∑_*i* < *j*_(*j* − *i*)/*N*, where the sum is over all *N* base pairs (*i, j*) in the secondary structure extracted from RNAview.

For benchmarking purposes, by removing short RNAs and RNAs for which RNAview yielded no base pairing information, we extracted a set of 180 PDB files with a total of 227 RNA chains from the datase [50] of 486 PDB files that have no discrepancies between the SEQRES and ATOM fields. For this benchmarking set, the left panel of Fig 13 shows a relatively high correlation between contact order and expected network degree—for instance, there is a correlation of 0.86 between 2D contact order and MS1 or MS2 network degree. Surprisingly, the correlation is generally higher when expected network degree is computed with respect to uniform probability (corresponding to energy model B with zero energy) rather than Boltzmann probability (corresponding to energy model C, i.e. Turner energy model). In the case of energy model C, the correlation is somewhat higher for move set MS1 rather than move set MS2.

The *number of native contacts* in a transitional protein structure is defined as the number of pairs of noncontiguous residues *i, j* that are in contact (i.e. close spatial proximity) in the native structure, usually meaning the X-ray structure [53]. The importance of this reaction coordinate for protein folding has been established in [54], where Best et al. analyze long equilibrium simulations of protein folding for more than 10 proteins using molecular dynamics trajectories from D.E. Shaw Research. It follows from Markov chain theory that the expected number of visitations of (transitional) structure *s* is the Boltzmann probability *p*(*s*) = exp(−*E*(*s*)/*RT*)/*Z* times the trajectory length, and hence the expected number of native contacts for RNA secondary structure formation can be defined by

$$\begin{array}{ccc} Q & = & \sum_{i<j}\sum_{s\in SS[1,n]}p\left(s\right)\cdot \left|\left\{\left(i,j\right):1\leq i<j\leq j,\left(i,j\right)\in s_{0}\right\}\right|=\sum_{i<j}\sum_{\left(i,j\right)\in s_{0}}p_{i,j} \end{array}$$ (95)

where ∣*s*_0_∣ denotes the number of base pairs in the native secondary structure *s*_0_, taken here to be the Rfam consensus structure used in benchmarking. In the right panel of Fig 13, we establish a relatively high correlation of 0.86 [resp. 0.84] between the expected number of native contacts for a collection 1904 RNA sequences and their consensus secondary structures from the Rfam 12.0 database and the uniform MS1 [resp. MS2] network degree. Again, it is worth pointing out that the slightly higher correlation of the MS1 measure over the MS2 measure.

RNA secondary structure folding kinetics remains a computationally difficult problem for RNA sequences of even moderate length, despite the availability of software to compute near-optimal folding pathways [7, 11, 13], compute population occupancy curves for coarse-grained models [14, 17, 18], and to repeatedly perform simulations of the Gillespie algorithm [5, 20–23, 30]. Our motivation in this article is to approach folding kinetics from a novel *network perspective*, where we show that network degree is moderately highly correlated with both *contact order* and the expected *number of native contacts*, both measures known to be correlated with experimentally measured protein folding kinetics. Despite the new algorithms of this paper and the existence of other software for RNA folding kinetics, it seems clear that significant progress in this field will require the a database of experimentally determined RNA folding rates, comparable to the database KineticDB containing experimentally determined folding rates for proteins [26].
